# Supplementary material for: HyperChIP: identification of hypervariable signals across ChIP-seq or ATAC-seq samples
Source: Genome Biol. 2022 Feb 28;23:62. doi: 10.1186/s13059-022-02627-9 (PMC8883642; doi:10.1186/s13059-022-02627-9)
Supplement: Supplementary file 1 — Additional file 1: Figure S1. Scatter plots showing various mean-variance trends associated with different data sets. Variance is shown at the log10 scale. Red lines depict the corresponding MVCs. Red points mark the 1000 regions with the largest scaled variances. Figure S2. Scatter plots of log10 scaled variances against observed mean signal intensities for different data sets. Red points mark the 1000 regions with the largest scaled variances. Figure S3. Applying other methods for ranking genomic regions and selecting HVRs. (a-c) Scatter plots showing the mean-variance trend (at proximal regions) associated with the H3K27ac ChIP-seq data set as well as the regions that are ranked in the top 1000 HVRs by each method (marked by red points). MAD, median absolute deviation; IQR, interquartile range. Figure S4. Identifying HVGs. We have separately identified HVGs for each data set in Table 1, by applying limma-trend to the corresponding RNA-seq data (see Methods in the main text for details). Scatter plots shown here demonstrate the modeling of the mean-variance relationships by limma-trend. Red points in each plot mark the identified HVGs. CPM, count per million; SD, standard deviation. Figure S5. Selecting a subset of genomic regions and using Winsorization for parameter estimation. (a) For the ATAC-seq and Pol II ChIP-seq data sets, bar plots showing the distributions of top-ranked proximal/distal HVRs along the range of mean intensities. For each data set, proximal and distal regions have been separately divided into 10 equally-sized groups based on the observed mean signal intensities. (b) Bar plots showing the distributions of top-ranked proximal/distal LVRs along the range of mean intensities. (c) d0 estimates resulting from different parameter estimation methods. Inf refers to positive infinity. Figure S6. The distributions of top-ranked proximal HVRs and LVRs associated with the Pol II ChIP-seq data set. The top-ranked LVRs form two clusters that are somewhat [file 13059_2022_2627_MOESM1_ESM.pdf]

Fig. S1

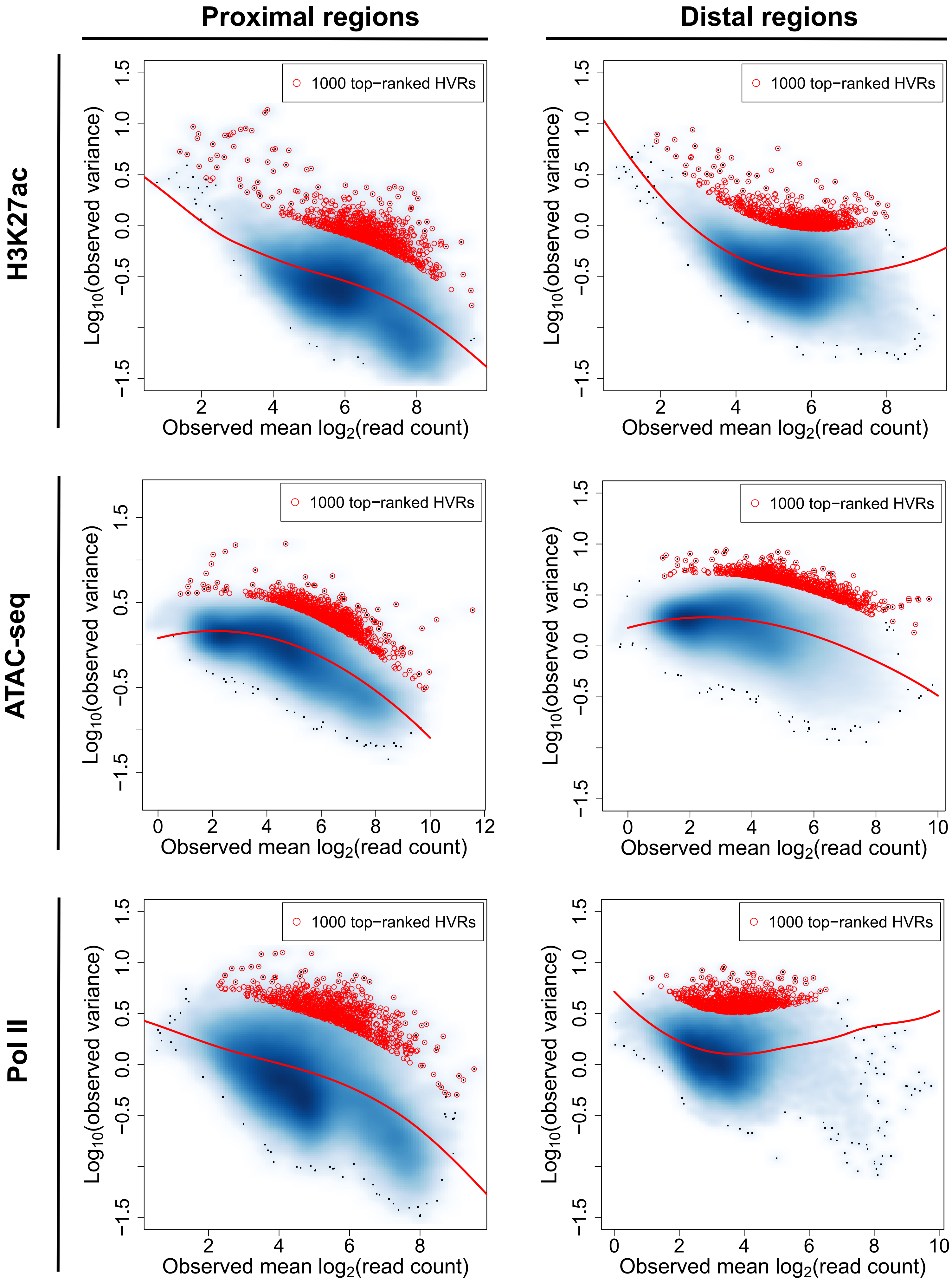

Proximal regions
Distal regions
H3K27ac
ATAC-seq
Pol II
1000 top−ranked HVRs
Log10(observed variance)
Observed mean log2(read count)

Figure S1. Scatter plots showing various mean-variance trends associated with different data sets. Variance is shown at the $\log_{10}$ scale. Red lines depict the corresponding MVCs. Red points mark the 1000 regions with the largest scaled variances.

Fig. S2

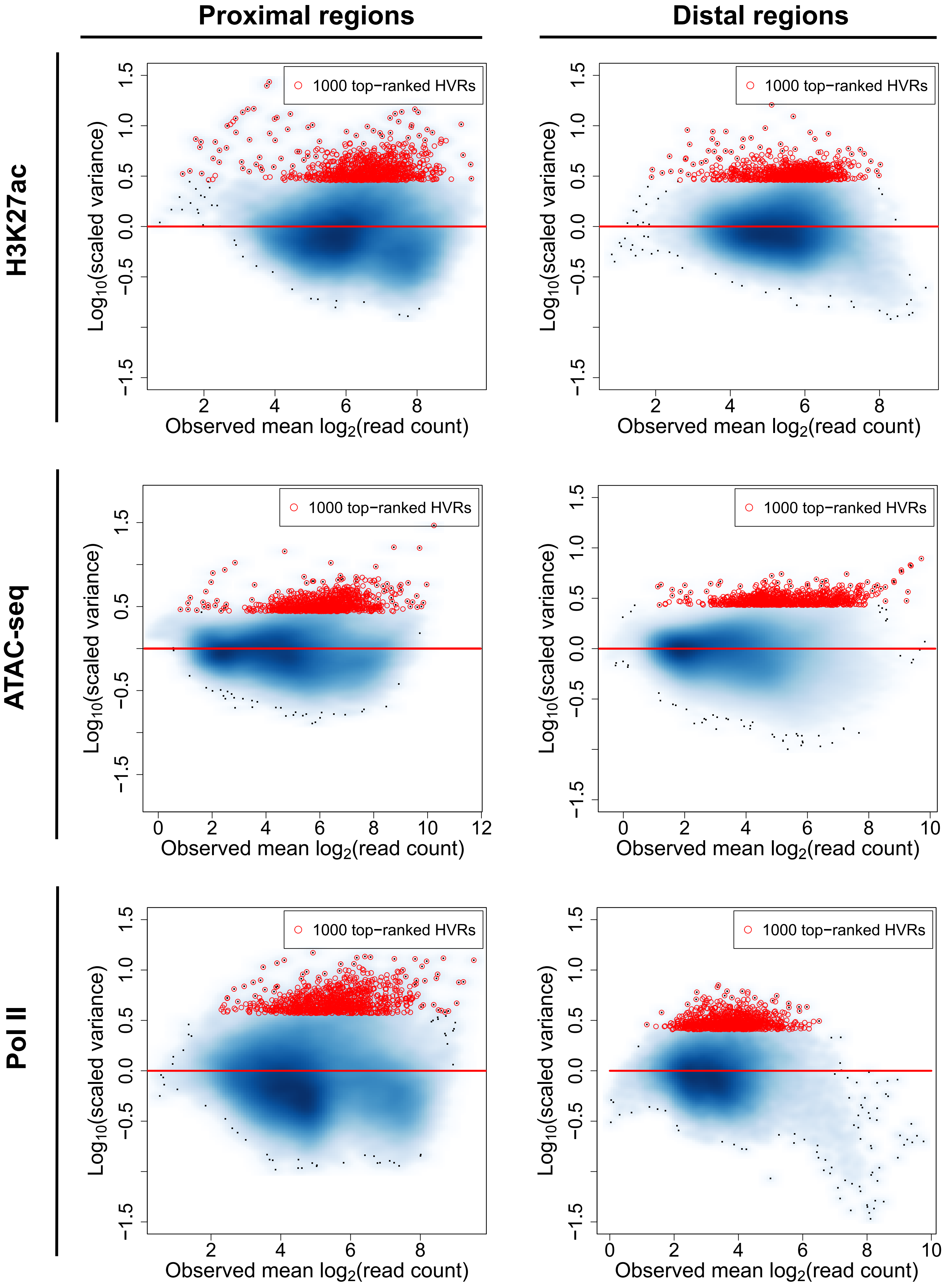

Proximal regions
Distal regions
H3K27ac
ATAC-seq
Pol II
1000 top−ranked HVRs
Log10(scaled variance)
Observed mean log2(read count)

Figure S2. Scatter plots of log_{10} scaled variances against observed mean signal intensities for different data sets. Red points mark the 1000 regions with the largest scaled variances.

Fig. S3

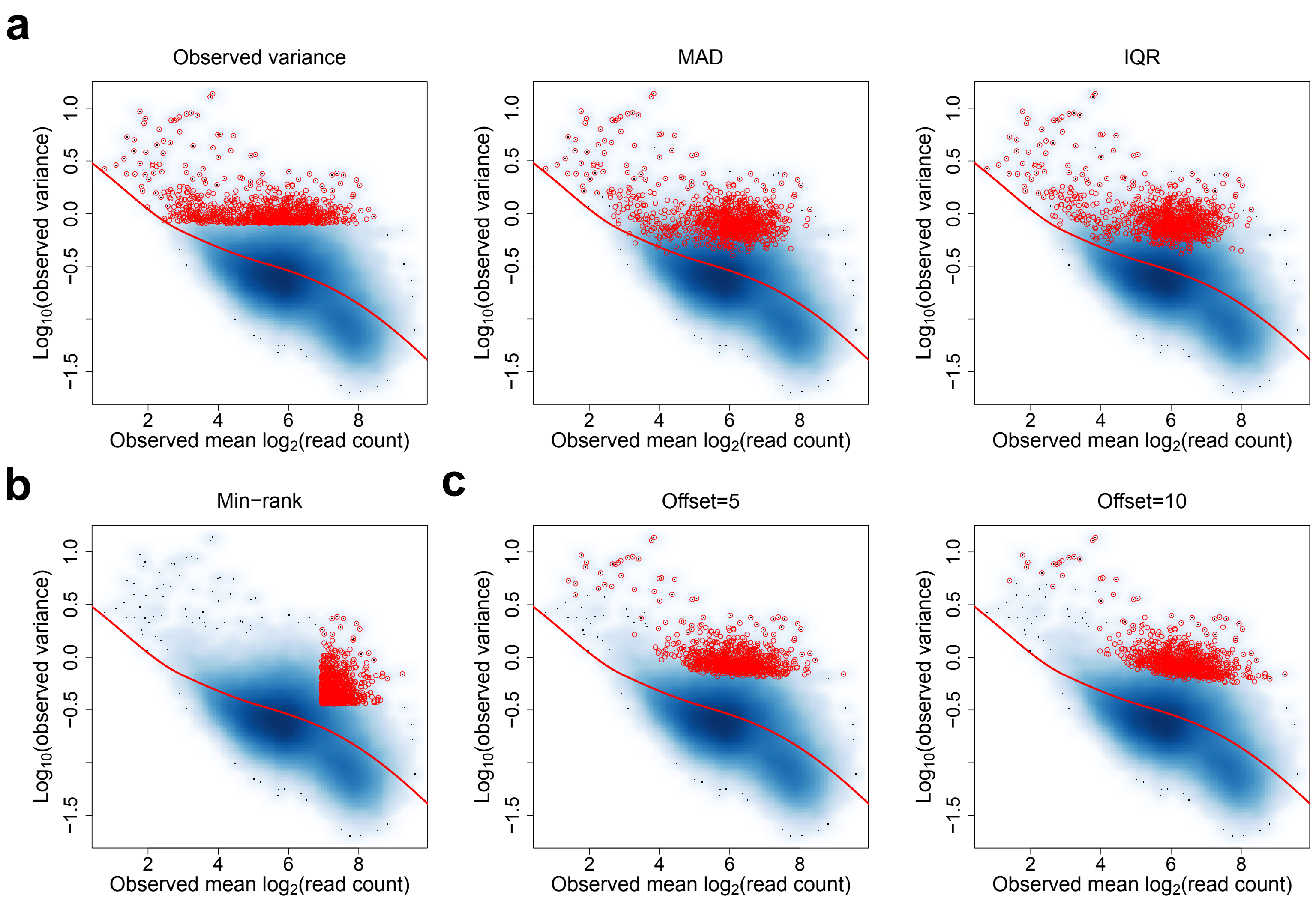
a
Observed variance
MAD
IQR
b
Min−rank
c
Offset=5
Offset=10
Log10(observed variance)
Observed mean log2(read count)
1.0
0.5
0.0
−0.5
−1.5
2
4
6
8

Figure S3. Applying other methods for ranking genomic regions and selecting HVRs. (a-c) Scatter plots showing the mean-variance trend (at proximal regions) associated with the H3K27ac ChIP-seq data set as well as the regions that are ranked in the top 1000 HVRs by each method (marked by red points). MAD, median absolute deviation; IQR, interquartile range.

Fig. S4

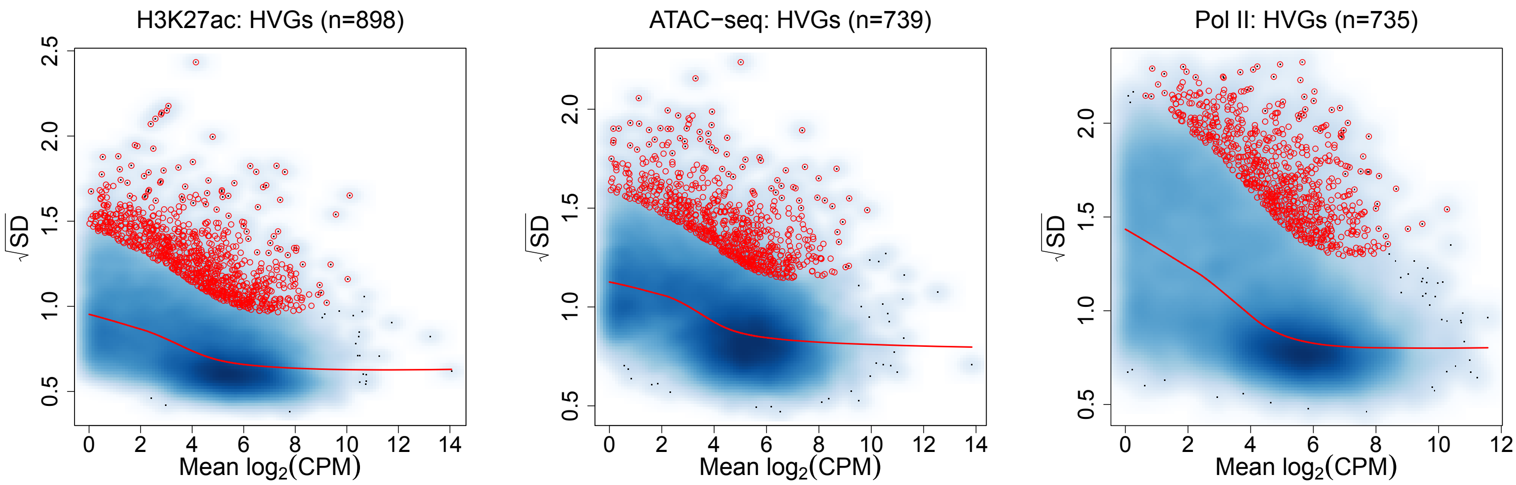

H3K27ac: HVGs (n=898)
ATAC−seq: HVGs (n=739)
Pol II: HVGs (n=735)
$\sqrt{SD}$
Mean $\log_2$(CPM)

Figure S4. Identifying HVGs. We have separately identified HVGs for each data set in Table 1, by applying limma-trend to the corresponding RNA-seq data (see Methods in the main text for details). Scatter plots shown here demonstrate the modeling of the mean-variance relationships by limma-trend. Red points in each plot mark the identified HVGs. CPM, count per million; SD, standard deviation.

Fig. S5

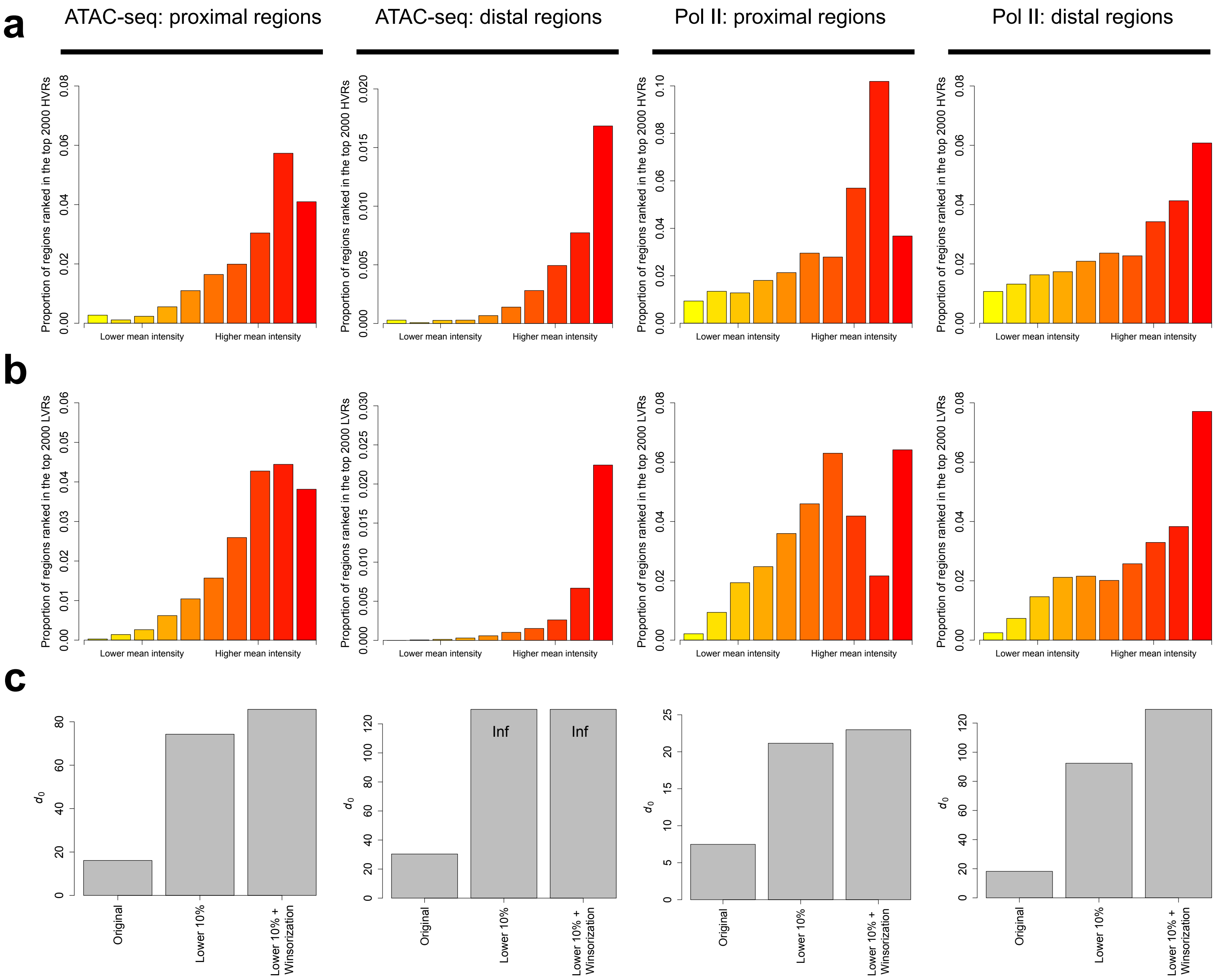
a
b
c
ATAC-seq: proximal regions
ATAC-seq: distal regions
Pol II: proximal regions
Pol II: distal regions
Proportion of regions ranked in the top 2000 HVRs
Proportion of regions ranked in the top 2000 LVRs
Lower mean intensity
Higher mean intensity
d0
Original
Lower 10%
Lower 10% + Winsorization
Inf
Inf

Figure S5. Selecting a subset of genomic regions and using Winsorization for parameter estimation. (a) For the ATAC-seq and Pol II ChIP-seq data sets, bar plots showing the distributions of top-ranked proximal/distal HVRs along the range of mean intensities. For each data set, proximal and distal regions have been separately divided into 10 equally-sized groups based on the observed mean signal intensities. **(b)** Bar plots showing the distributions of top-ranked proximal/distal LVRs along the range of mean intensities. **(c)** d_0 estimates resulting from different parameter estimation methods. Inf refers to positive infinity.

Fig. S6

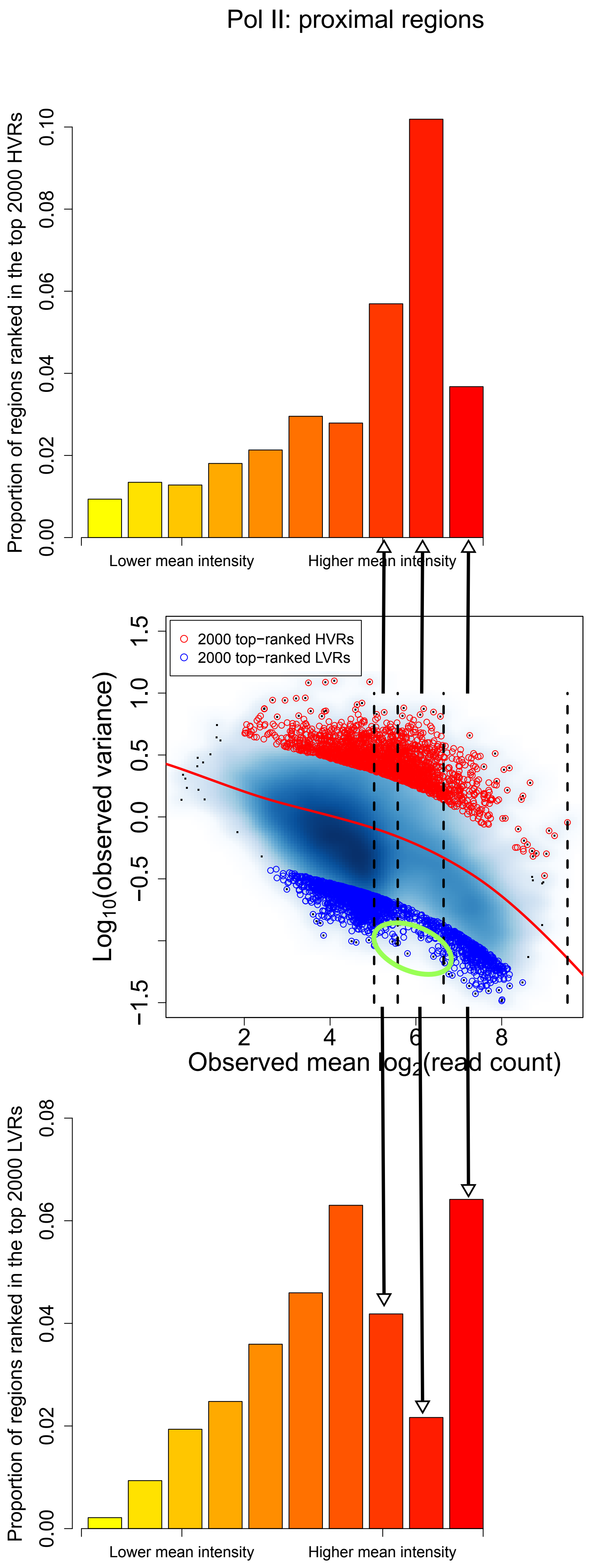
Pol II: proximal regions
Proportion of regions ranked in the top 2000 HVRs
0.00
0.02
0.04
0.06
0.08
0.10
Lower mean intensity
Higher mean intensity
2000 top−ranked HVRs
2000 top−ranked LVRs
Log10(observed variance)
−1.5
−0.5
0.0
0.5
1.0
1.5
2
4
6
8
Observed mean log2(read count)
Proportion of regions ranked in the top 2000 LVRs
0.00
0.02
0.04
0.06
0.08
Lower mean intensity
Higher mean intensity

Figure S6. The distributions of top-ranked proximal HVRs and LVRs associated with the Pol II ChIP-seq data set. The top-ranked LVRs form two clusters that are somewhat separated from one another in the mean-variance scatter plot, owing to a gap (indicated by the area circled in green) largely corresponding to the 70th to 90th percentile of mean intensities. As a result, the proportion of the LVRs dips at the corresponding two groups of regions, leading to a bimodal distribution profile as well as a rise in the HVR proportion that is more dramatic compared to the other two data sets.

Fig. S7

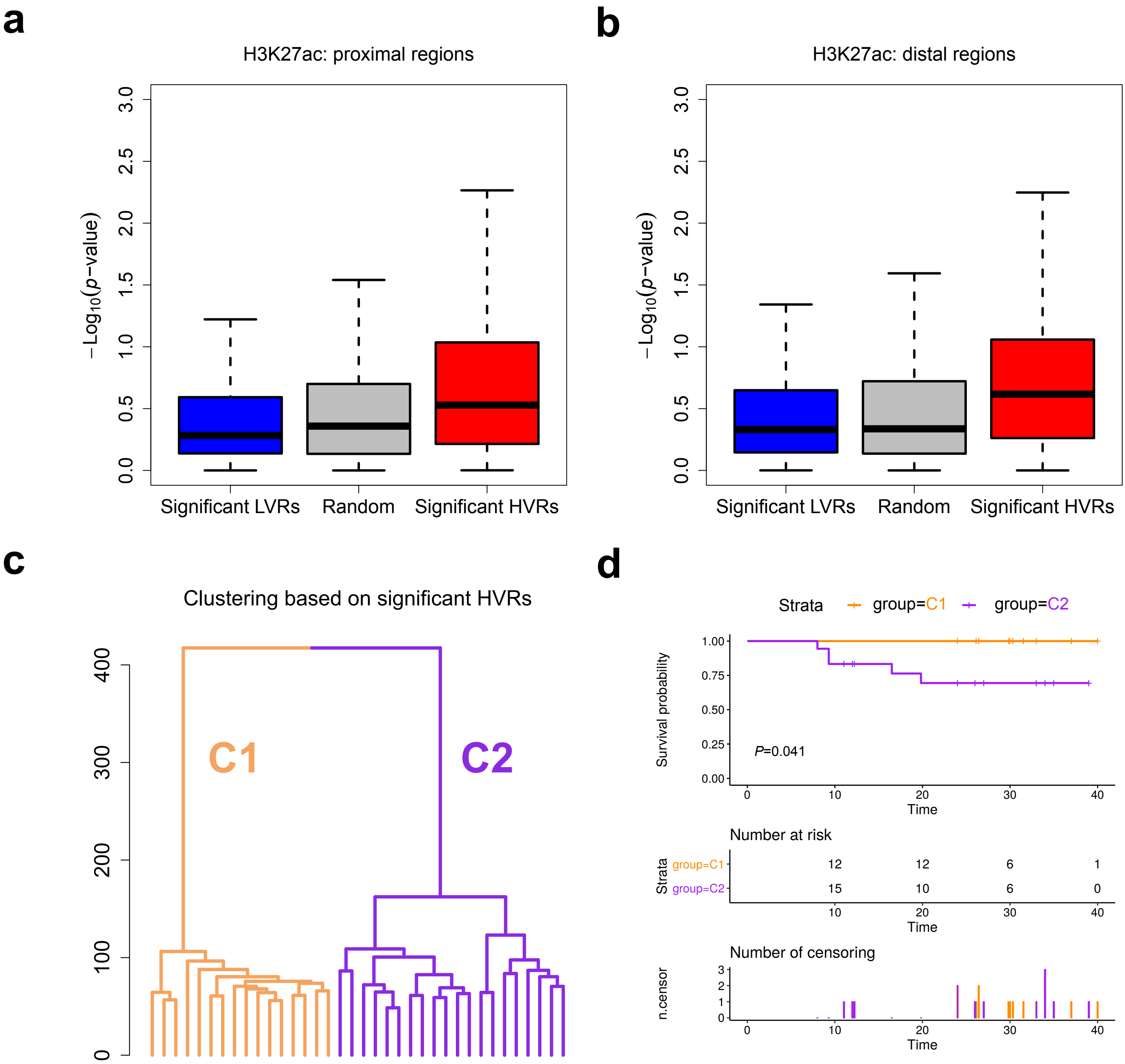
a
H3K27ac: proximal regions
−Log10(p−value)
0.0
0.5
1.0
1.5
2.0
2.5
3.0
Significant LVRs
Random
Significant HVRs
b
H3K27ac: distal regions
−Log10(p−value)
0.0
0.5
1.0
1.5
2.0
2.5
3.0
Significant LVRs
Random
Significant HVRs
c
Clustering based on significant HVRs
0
100
200
300
400
C1
C2
d
Strata
group=C1
group=C2
Survival probability
1.00
0.75
0.50
0.25
0.00
P=0.041
0
10
20
30
40
Time
Number at risk
Strata
group=C1
group=C2
12
12
6
1
15
10
6
0
10
20
30
40
Time
Number of censoring
n.censor
3
2
1
0
0
10
20
30
40
Time

Figure S7. Evaluating the prognostic associations of different genomic regions. (a, b) Proximal/distal HVRs are more significantly associated with the survival time of patients than proximal/distal LVRs and randomly selected proximal/distal peak regions. Results shown here are based on the H3K27ac ChIP-seq data set. The *p*-values are derived by separately performing a Cox regression on the H3K27ac level in each region. **(c)** Dendrogram showing the hierarchical clustering of the patients based on the proximal and distal HVRs. The patients are classified into two sub-groups, labeled C1 and C2. **(d)** There is a significant survival difference between C1 and C2.

Fig. S8

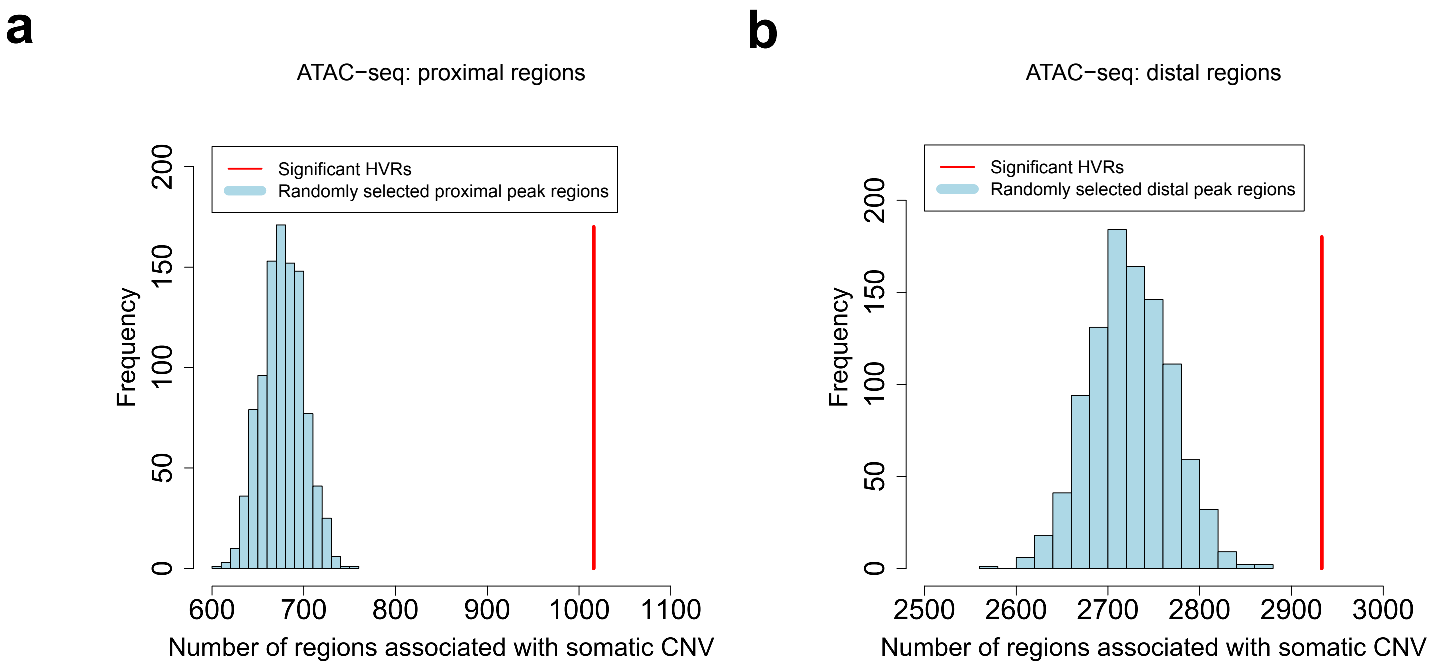

a
ATAC−seq: proximal regions
Significant HVRs
Randomly selected proximal peak regions
Frequency
0
50
100
150
200
600
700
800
900
1000
1100
Number of regions associated with somatic CNV
b
ATAC−seq: distal regions
Significant HVRs
Randomly selected distal peak regions
Frequency
0
50
100
150
200
2500
2600
2700
2800
2900
3000
Number of regions associated with somatic CNV

Figure S8. HVRs have a significant association with somatic copy number variation (CNV). (a, b) Proximal/distal HVRs are more likely to be associated with somatic CNV than randomly selected proximal/distal peak regions. Results shown here are based on the ATAC-seq data set. For this data set, the genomic segments with somatic CNV in at least one patient together occupied almost the whole genome (>95%). We therefore considered a region as associated with somatic CNV only if it overlapped CNV segments in more than 5 patients. 1,000 random simulations were performed separately for proximal and distal peak regions. In each time, we randomly selected the same number of proximal/distal peak regions as that of the proximal/distal HVRs.

Fig. S9

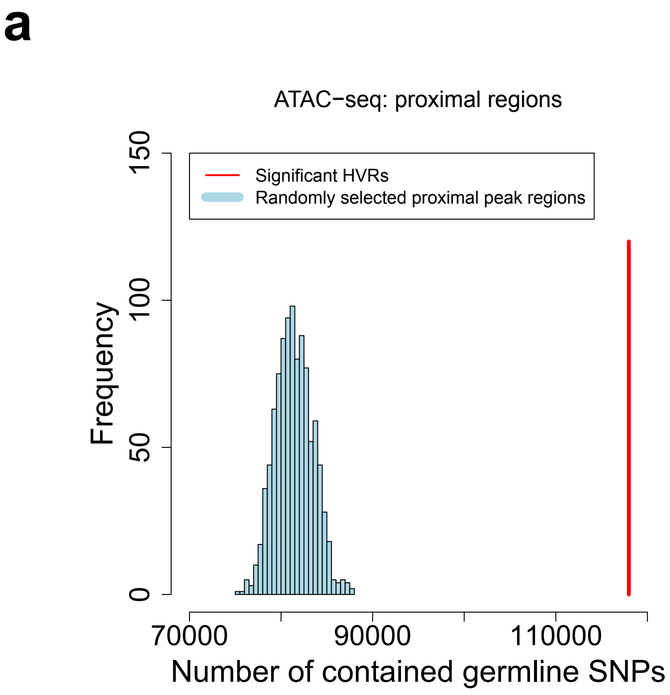
a
ATAC−seq: proximal regions
Significant HVRs
Randomly selected proximal peak regions
Frequency
0
50
100
150
70000
90000
110000
Number of contained germline SNPs

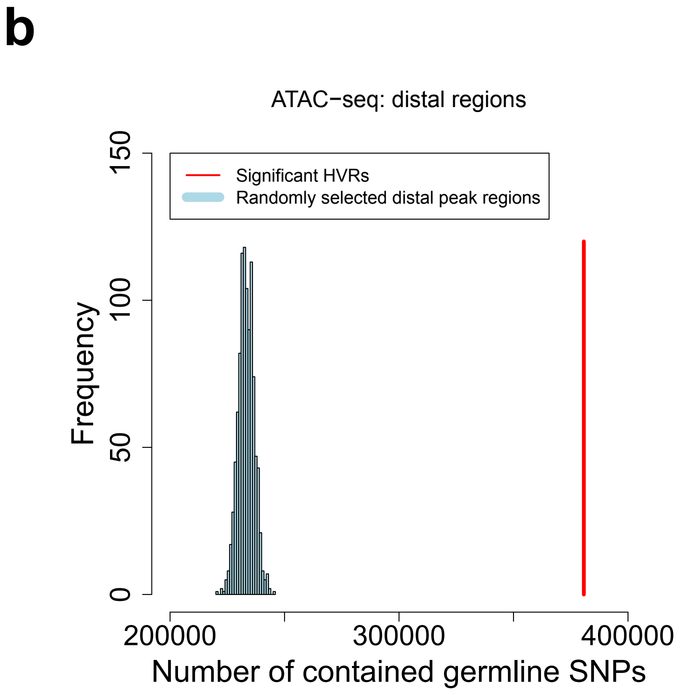
b
ATAC−seq: distal regions
Significant HVRs
Randomly selected distal peak regions
Frequency
0
50
100
150
200000
300000
400000
Number of contained germline SNPs

Figure S9. HVRs contain significantly more germline SNPs than by chance. (a) Proximal HVRs identified for the ATAC-seq data set are enriched with germline SNPs. We have performed 1,000 times of random simulation. In each time, a set of proximal peak regions matching the number of the HVRs has been randomly selected. **(b)** Distal HVRs are enriched with germline SNPs as well.

Fig. S10

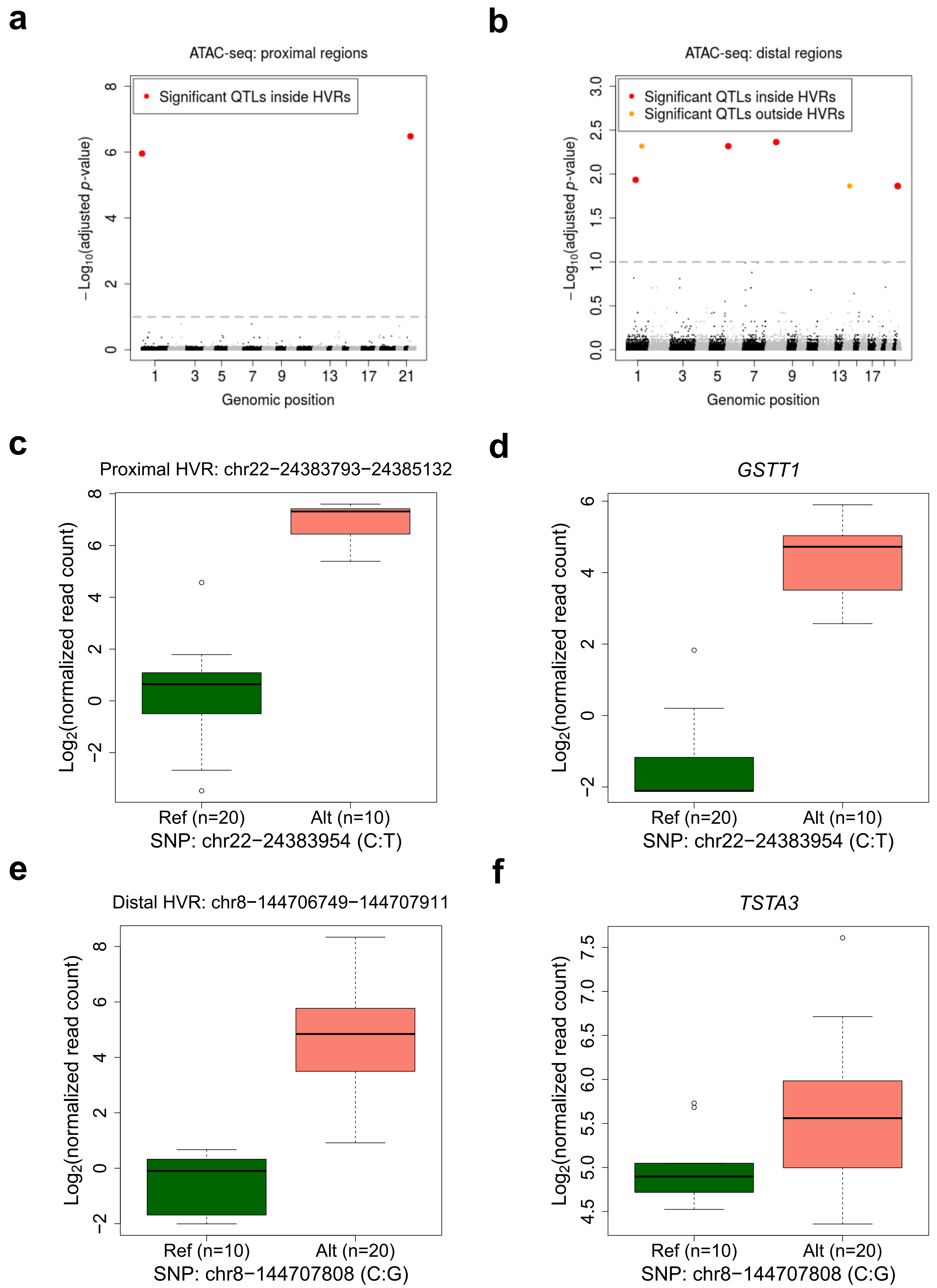
a
ATAC-seq: proximal regions
Significant QTLs inside HVRs
−Log10(adjusted p-value)
Genomic position
b
ATAC-seq: distal regions
Significant QTLs inside HVRs
Significant QTLs outside HVRs
−Log10(adjusted p-value)
Genomic position
c
Proximal HVR: chr22−24383793−24385132
Log2(normalized read count)
Ref (n=20)
Alt (n=10)
SNP: chr22−24383954 (C:T)
d
GSTT1
Log2(normalized read count)
Ref (n=20)
Alt (n=10)
SNP: chr22−24383954 (C:T)
e
Distal HVR: chr8−144706749−144707911
Log2(normalized read count)
Ref (n=10)
Alt (n=20)
SNP: chr8−144707808 (C:G)
f
TSTA3
Log2(normalized read count)
Ref (n=10)
Alt (n=20)
SNP: chr8−144707808 (C:G)

Figure S10. Association between QTLs and HVRs. (a, b) Identifying QTLs among the germline SNPs located within ATAC-seq peak regions. Each (BH-adjusted) *p*-value assesses the statistical significance of the association between the genotype of a SNP and the ATAC-seq signal in the peak region containing it. **(c)** Box plots showing the ATAC-seq signals associated with different genotypes of the most significant QTL, which is located within a proximal HVR. Ref and Alt refer to the reference genotype and the alternative one, respectively. **(d)** Box plots showing the RNA-seq signals of the downstream gene of the proximal HVR. **(e)** Box plots showing the ATAC-seq signals associated with different genotypes of the most significant distal QTL, which is also located within an HVR. **(f)** Box plots showing the RNA-seq signals of the gene nearest to the distal HVR.

Fig. S11

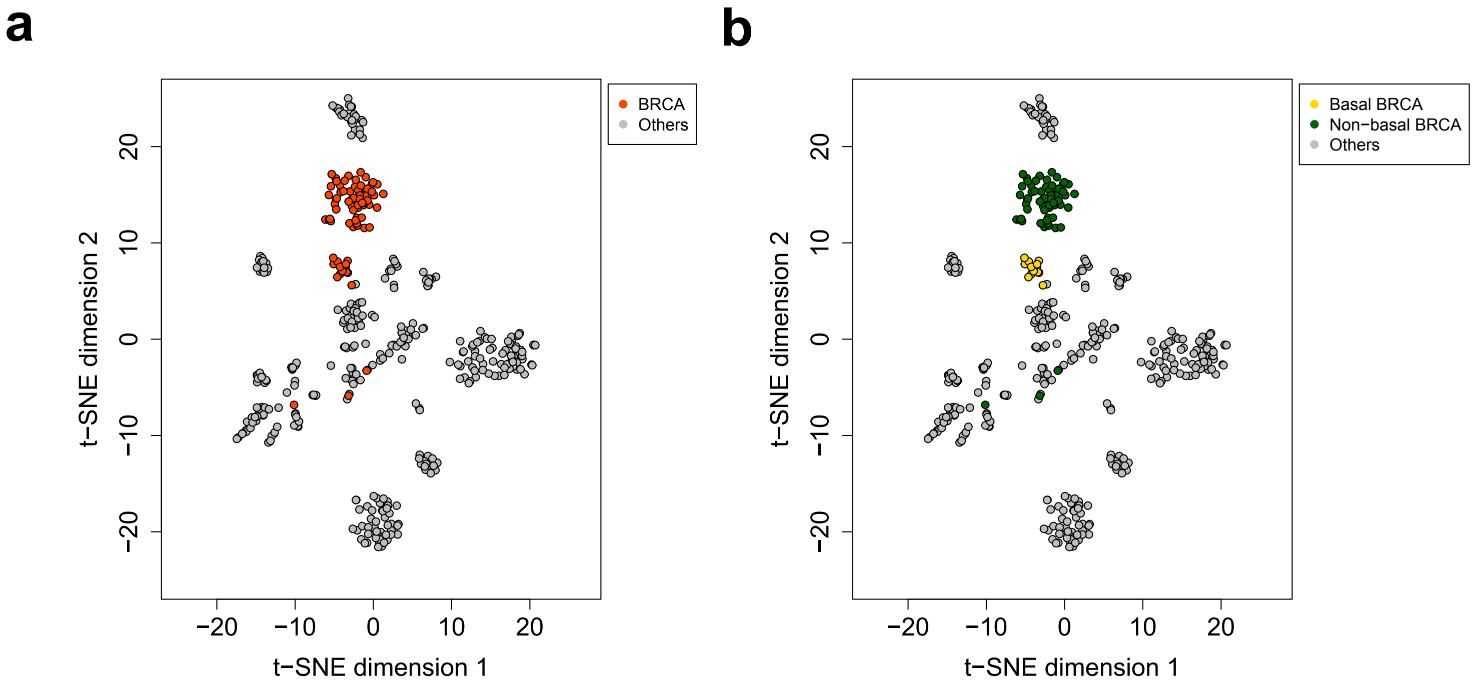
a
BRCA
Others
t−SNE dimension 2
t−SNE dimension 1
20
10
0
−10
−20
b
Basal BRCA
Non−basal BRCA
Others
t−SNE dimension 2
t−SNE dimension 1

Figure S11. For the TCGA pan-cancer ATAC-seq data set, two-dimensional t-SNE plots showing the distribution of BRCA patients. These patients are comprised of 14 basal and 61 non-basal cases.

Fig. S12

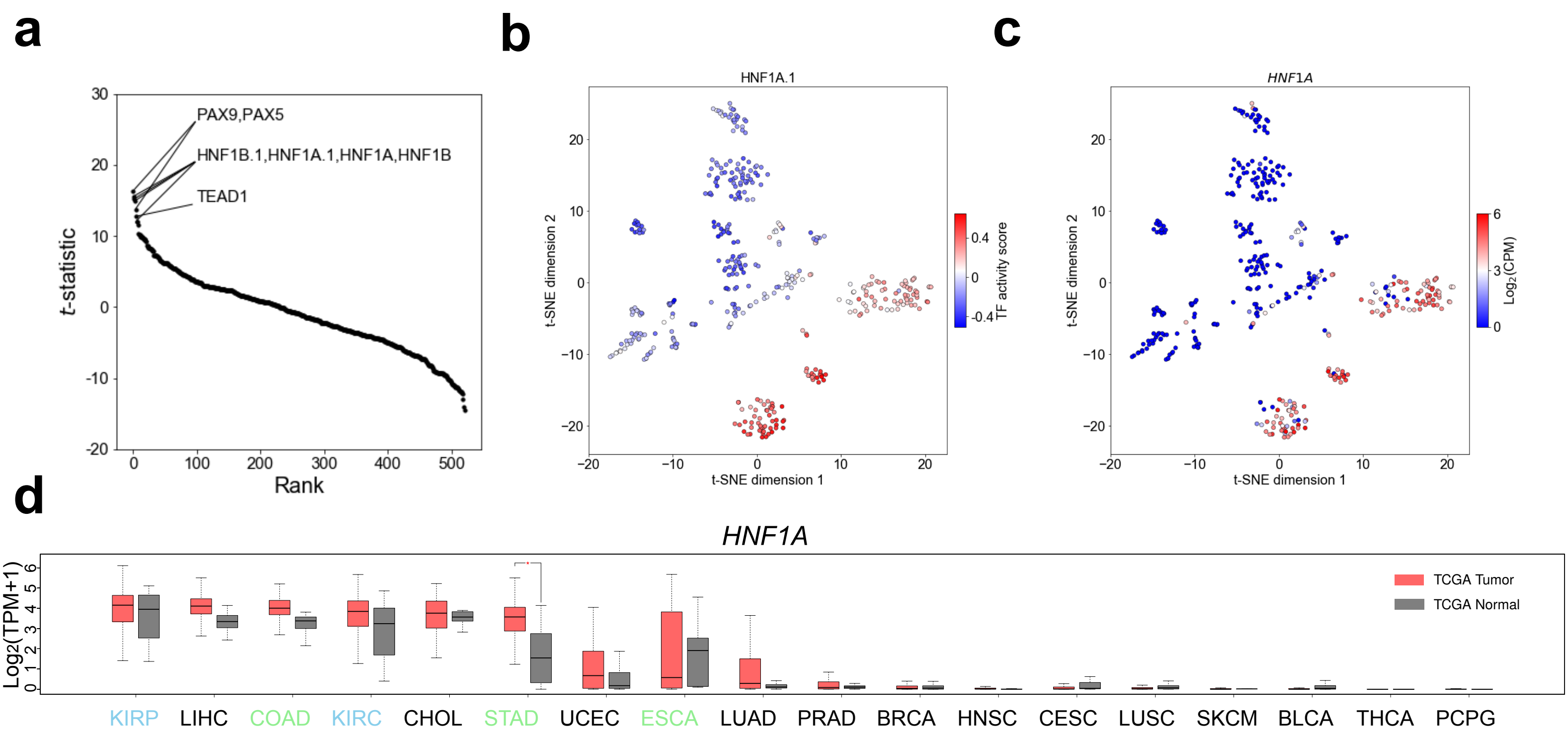
a
b
c
d
PAX9,PAX5
HNF1B.1,HNF1A.1,HNF1A,HNF1B
TEAD1
t-statistic
Rank
HNF1A.1
TF activity score
t-SNE dimension 1
t-SNE dimension 2
HNF1A
Log2(CPM)
HNF1A
Log2(TPM+1)
TCGA Tumor
TCGA Normal
KIRP
LIHC
COAD
KIRC
CHOL
STAD
UCEC
ESCA
LUAD
PRAD
BRCA
HNSC
CESC
LUSC
SKCM
BLCA
THCA
PCPG

Figure S12. *HNF1A* is identified as a top-ranked TF for the kidney carcinoma class. (a) Plotting the *t*-statistics of all motifs against their rankings in the identification of TFs specific to the kidney carcinoma class. **(b)** Mapping the TF activity scores associated with the HNF1A.1 motif to the t-SNE plot. **(c)** Mapping the expression levels of the *HNF1A* gene to the t-SNE plot. **(d)** Box plots showing the expression of *HNF1A* in a larger TCGA cohort of patients. TPM, transcripts per million.

Fig. S13

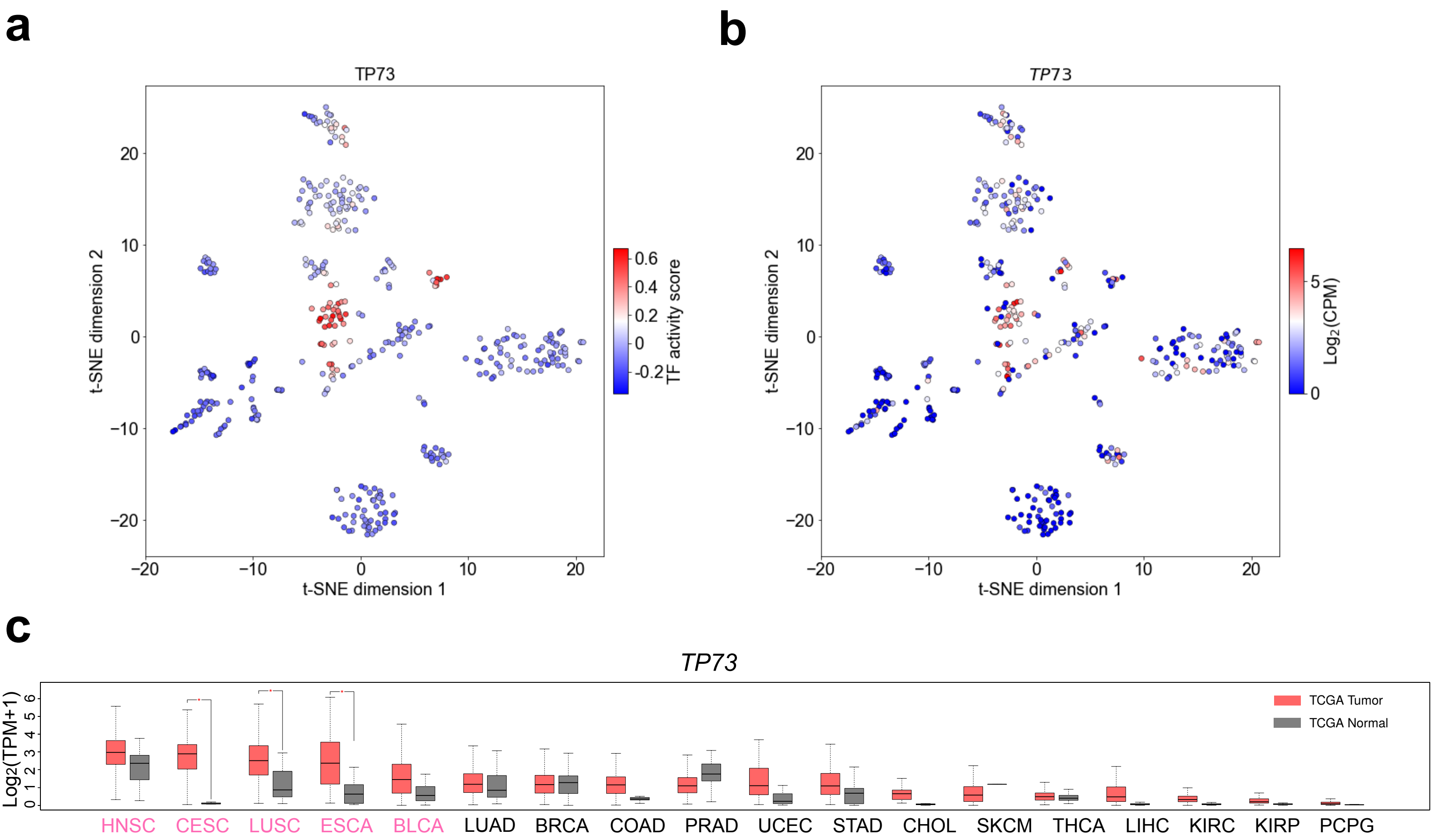
a
b
c
TP73
t-SNE dimension 1
t-SNE dimension 2
TF activity score
Log2(CPM)
Log2(TPM+1)
TCGA Tumor
TCGA Normal
HNSC
CESC
LUSC
ESCA
BLCA
LUAD
BRCA
COAD
PRAD
UCEC
STAD
CHOL
SKCM
THCA
LIHC
KIRC
KIRP
PCPG

Figure S13. *TP73* is identified as a top-ranked TF for the SC class. (a) Mapping the TF activity scores associated with the TP73 motif to the t-SNE plot. **(b)** Mapping the expression levels of the *TP73* gene to the t-SNE plot. **(c)** Box plots showing the expression of *TP73* in the larger TCGA cohort.

Fig. S14

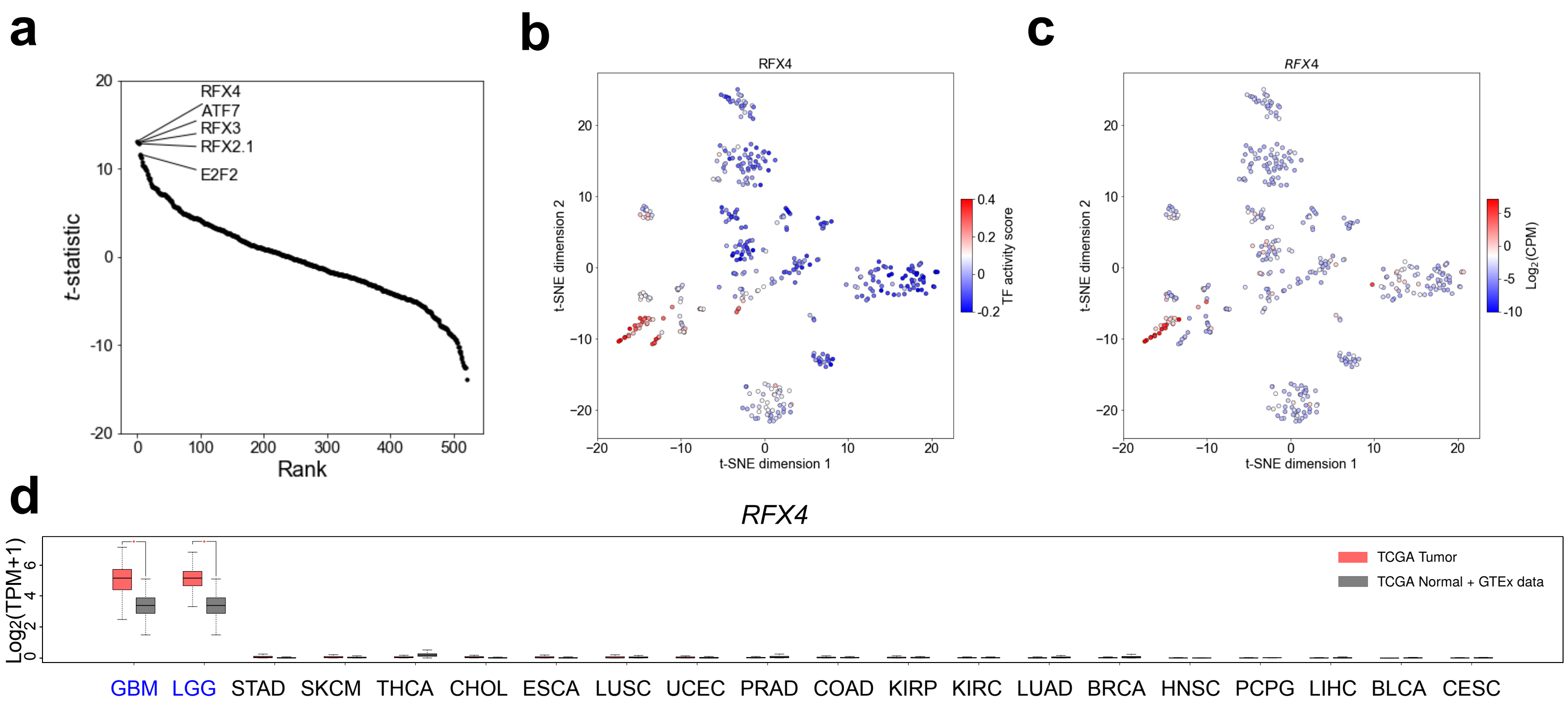

a
RFX4
ATF7
RFX3
RFX2.1
E2F2
t-statistic
Rank
b
RFX4
t-SNE dimension 2
t-SNE dimension 1
TF activity score
c
RFX4
t-SNE dimension 2
t-SNE dimension 1
Log2(CPM)
d
RFX4
Log2(TPM+1)
TCGA Tumor
TCGA Normal + GTEx data
GBM
LGG
STAD
SKCM
THCA
CHOL
ESCA
LUSC
UCEC
PRAD
COAD
KIRP
KIRC
LUAD
BRCA
HNSC
PCPG
LIHC
BLCA
CESC

Figure S14. *RFX4* ranks first among the brain cancer class-specific TFs. (a) Plotting the *t*-statistics of all motifs against their rankings in the identification of TFs specific to the brain cancer class. **(b)** Mapping the TF activity scores associated with the RFX4 motif to the t-SNE plot. **(c)** Mapping the expression levels of the *RFX4* gene to the t-SNE plot. **(d)** Box plots showing the expression of *RFX4* in the larger TCGA cohort as well as in 3,006 RNA-seq samples of normal individuals provided by the GTEx (Genotype-Tissue Expression) project (https://gtexportal.org/home/). We involved the GTEx data because RNA-seq samples for matched normal tissues of GBM and LGG were missing in the TCGA program.

Fig. S15

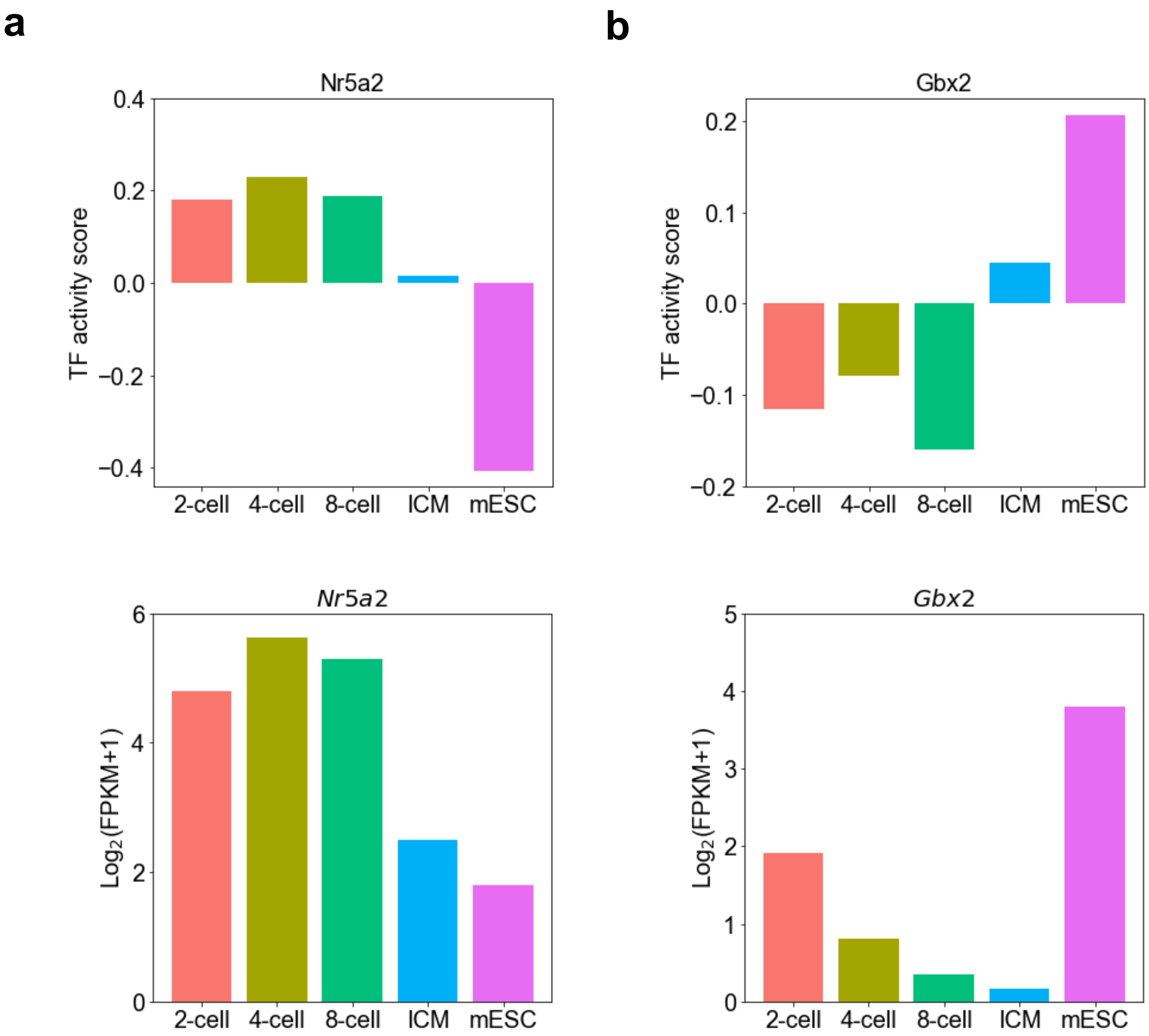

a
b
Nr5a2
TF activity score
0.4
0.2
0.0
−0.2
−0.4
2-cell
4-cell
8-cell
ICM
mESC
Gbx2
0.2
0.1
0.0
−0.1
−0.2
Nr5a2
Log2(FPKM+1)
6
4
2
0
Gbx2
5
4
3
2
1
0

Figure S15. Examples of stage-specific regulators identified from the mouse ATAC-seq data set. (a, b) Bar plots showing the TF activity scores and expression levels of (a) *Nr5a2* and (b) *Gbx2*, which are top-ranked TFs associated with the early and late stages, respectively. TF activity scores have been averaged across biological replicates for each individual cell stage.

Fig. S16

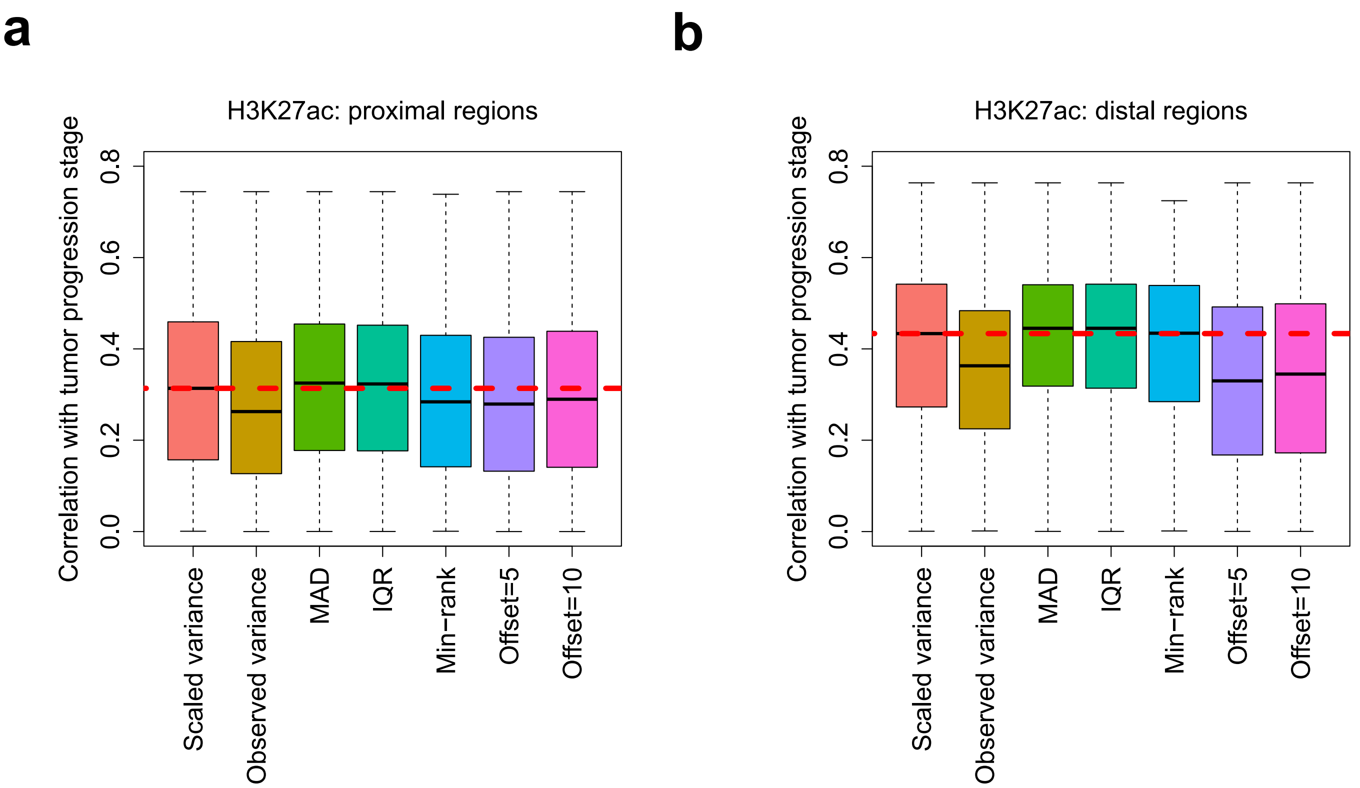
a
b
H3K27ac: proximal regions
H3K27ac: distal regions
Correlation with tumor progression stage
0.0
0.2
0.4
0.6
0.8
Scaled variance
Observed variance
MAD
IQR
Min−rank
Offset=5
Offset=10

Figure S16. Evaluating the correlations of HVRs identified by different methods with tumor progression stage. (a, b) Results shown here are based on the LUAD H3K27ac ChIP-seq data set. For each method, the same number of top-ranked proximal/distal HVRs as identified by HyperChIP are selected. The red dotted line in each plot indicates the median correlation of the HVRs identified by HyperChIP.

Fig. S17

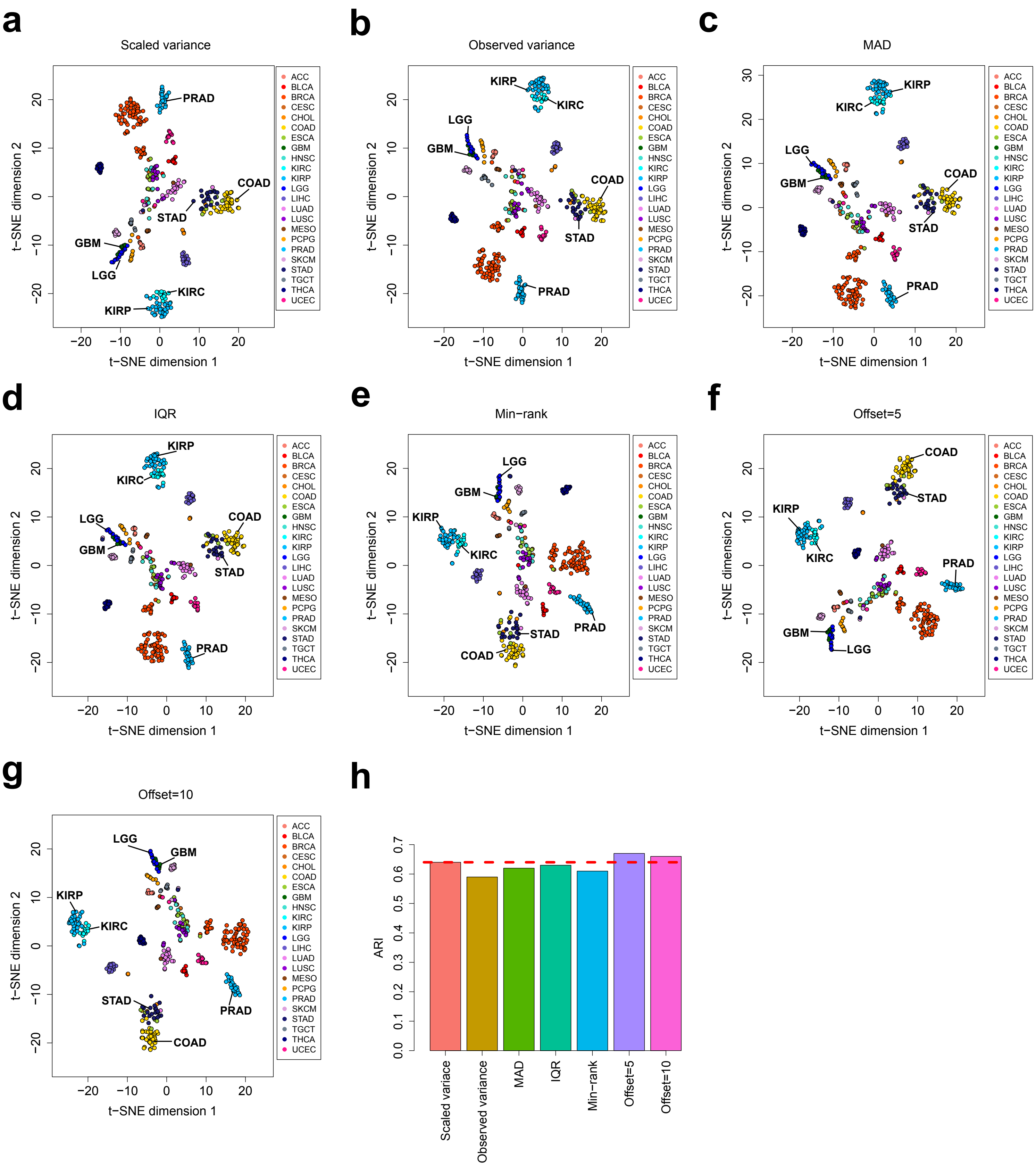
a
Scaled variance
PRAD
COAD
STAD
GBM
LGG
KIRC
KIRP
t−SNE dimension 1
t−SNE dimension 2
ACC
BLCA
BRCA
CESC
CHOL
COAD
ESCA
GBM
HNSC
KIRC
KIRP
LGG
LIHC
LUAD
LUSC
MESO
PCPG
PRAD
SKCM
STAD
TGCT
THCA
UCEC
b
Observed variance
KIRP
KIRC
LGG
GBM
COAD
STAD
PRAD
c
MAD
KIRP
KIRC
LGG
GBM
COAD
STAD
PRAD
d
IQR
KIRP
KIRC
LGG
GBM
COAD
STAD
PRAD
e
Min−rank
LGG
GBM
KIRP
KIRC
STAD
PRAD
COAD
f
Offset=5
COAD
STAD
KIRP
KIRC
PRAD
GBM
LGG
g
Offset=10
LGG
GBM
KIRP
KIRC
STAD
PRAD
COAD
h
ARI
Scaled variace
Observed variance
MAD
IQR
Min−rank
Offset=5
Offset=10

Figure S17. Applying different methods to the pan-cancer ATAC-seq data set. (a-g) Two-dimensional t-SNE plots generated by different methods. For each method, the same number of top-ranked proximal/distal HVRs as identified by HyperChIP were used for the downstream PCA and t-SNE analysis (see Methods in the main text). **(h)** Bar plot showing the ARI values achieved by different methods in classifying all samples. For each method, we performed a hierarchical clustering of all samples based on the same principal components as used in the t-SNE analysis. The samples were then classified into 23 sub-groups based on the resulting hierarchical tree, and the corresponding ARI assessed the agreement of this classification with the cancer type labels. The red dotted line indicates the ARI value achieved by HyperChIP.

Fig. S18

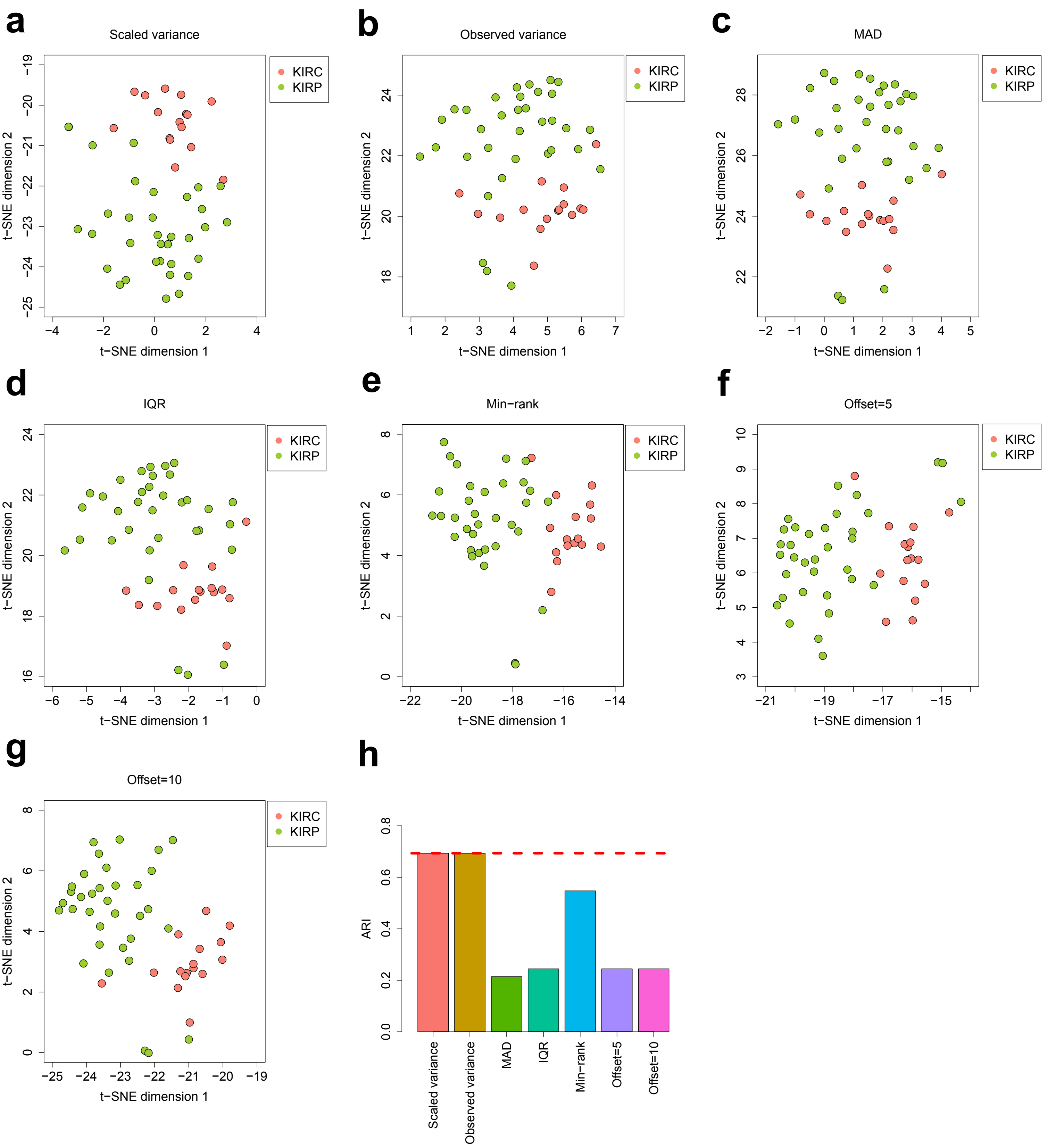
a
Scaled variance
b
Observed variance
c
MAD
d
IQR
e
Min−rank
f
Offset=5
g
Offset=10
h
KIRC
KIRP
t−SNE dimension 1
t−SNE dimension 2
ARI
Scaled variance
Observed variance
MAD
IQR
Min−rank
Offset=5
Offset=10

Figure S18. Evaluating the ability of different methods to distinguish between the KIRC and KIRP cancer types. (a-g) Zooming in on the t-SNE plots to more clearly present the distributions of KIRC and KIRP samples. **(h)** Bar plot showing the ARI values achieved by different methods in classifying KIRC and KIRP samples. For each method, we performed a hierarchical clustering of KIRC and KIRP samples and classified them into two sub-groups based on the resulting hierarchical tree.

Fig. S19

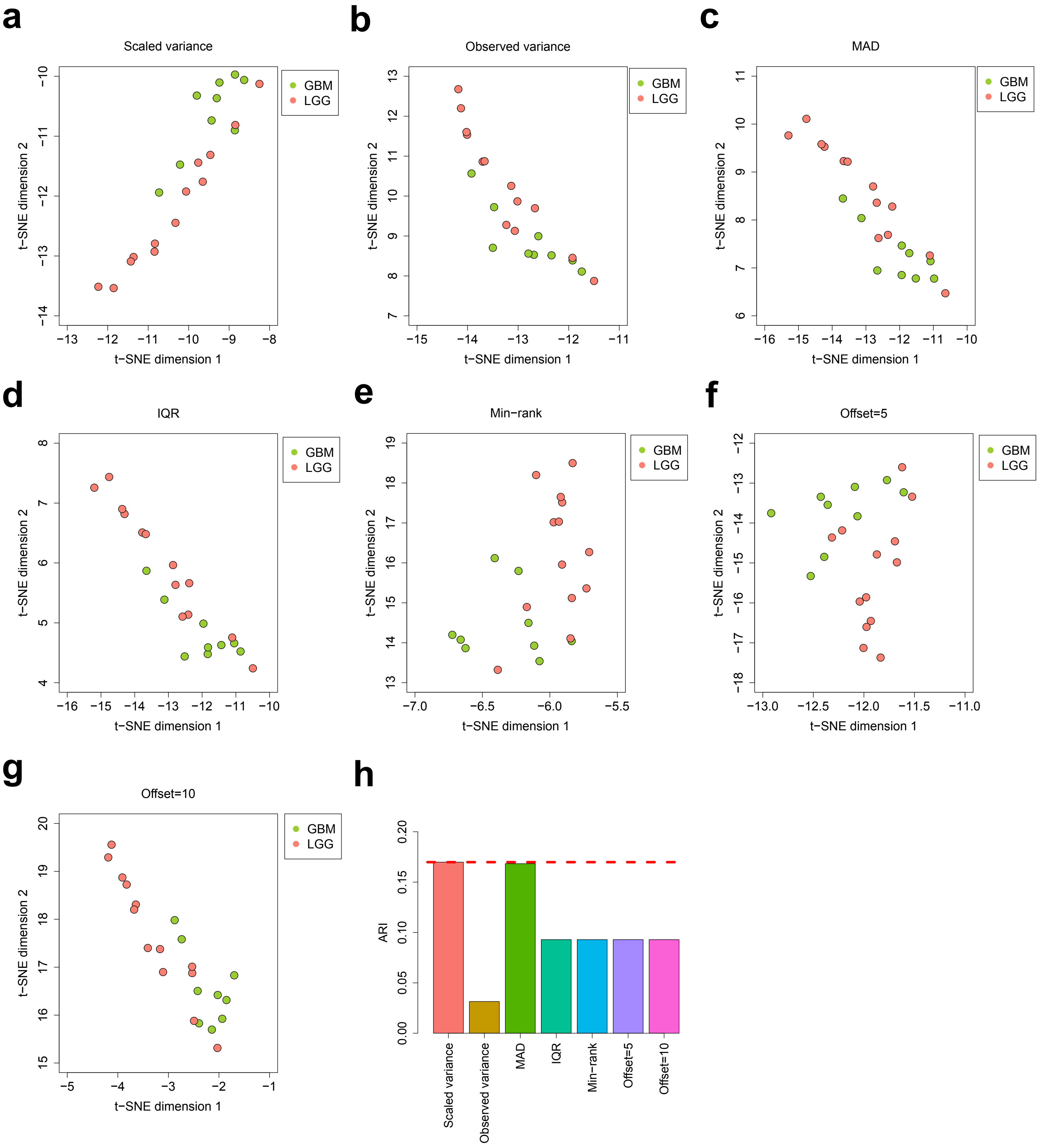

a
Scaled variance
t−SNE dimension 1
t−SNE dimension 2
GBM
LGG
b
Observed variance
t−SNE dimension 1
t−SNE dimension 2
GBM
LGG
c
MAD
t−SNE dimension 1
t−SNE dimension 2
GBM
LGG
d
IQR
t−SNE dimension 1
t−SNE dimension 2
GBM
LGG
e
Min−rank
t−SNE dimension 1
t−SNE dimension 2
GBM
LGG
f
Offset=5
t−SNE dimension 1
t−SNE dimension 2
GBM
LGG
g
Offset=10
t−SNE dimension 1
t−SNE dimension 2
GBM
LGG
h
ARI
Scaled variance
Observed variance
MAD
IQR
Min−rank
Offset=5
Offset=10

Figure S19. Evaluating the ability of different methods to distinguish between the GBM and LGG cancer types. (a-g) Zooming in on the t-SNE plots to more clearly present the distributions of GBM and LGG samples. **(h)** Bar plot showing the ARI values achieved by different methods in classifying GBM and LGG samples. For each method, we performed a hierarchical clustering of GBM and LGG samples and classified them into two sub-groups based on the resulting hierarchical tree.

Fig. S20

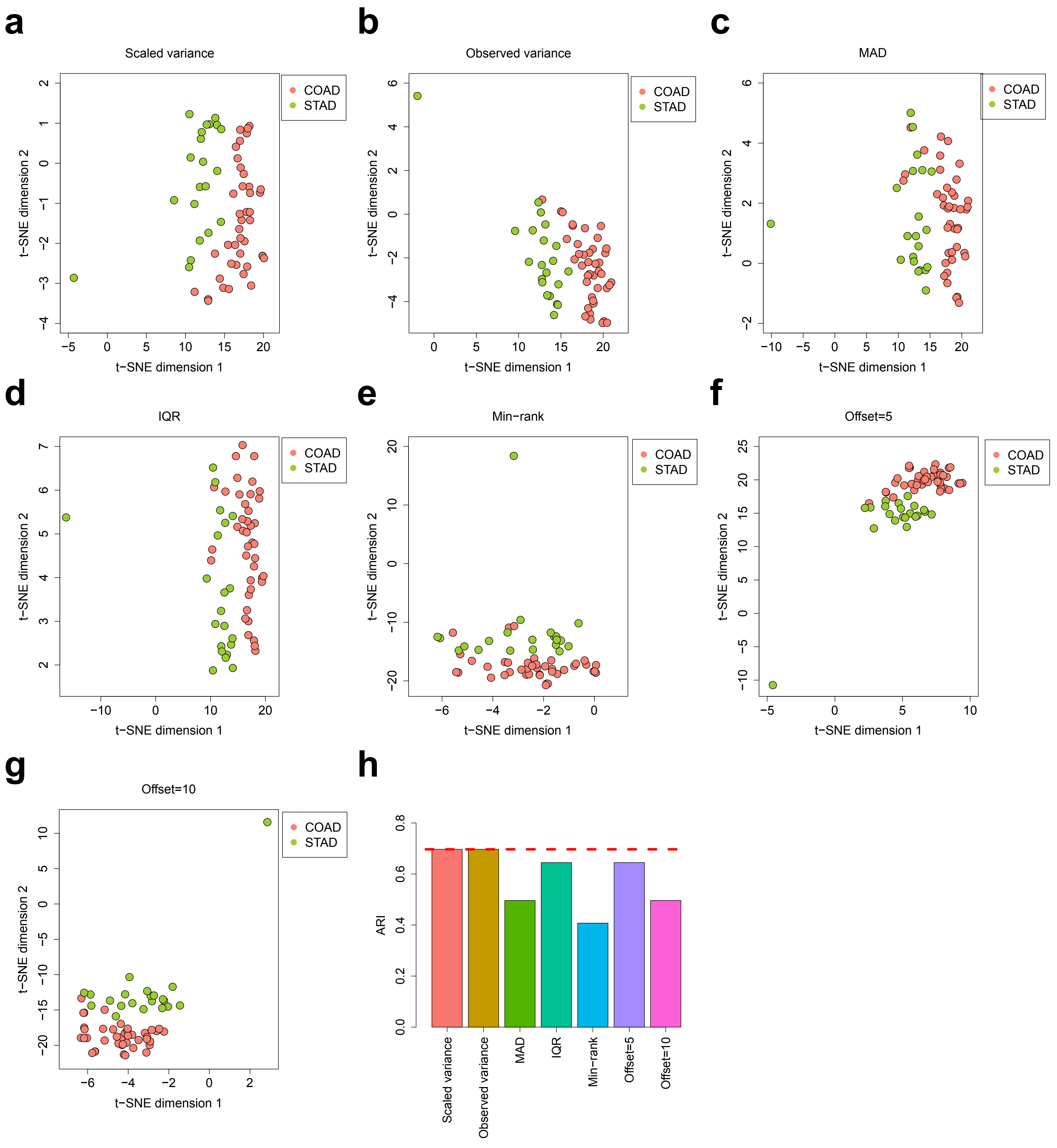
a
Scaled variance
b
Observed variance
c
MAD
d
IQR
e
Min−rank
f
Offset=5
g
Offset=10
h
COAD
STAD
t−SNE dimension 1
t−SNE dimension 2
ARI
Scaled variance
Observed variance
MAD
IQR
Min−rank
Offset=5
Offset=10

Figure S20. Evaluating the ability of different methods to distinguish between the COAD and STAD cancer types. (a-g) Zooming in on the t-SNE plots to more clearly present the distributions of COAD and STAD samples. **(h)** Bar plot showing the ARI values achieved by different methods in classifying COAD and STAD samples. For each method, we performed a hierarchical clustering of COAD and STAD samples and classified them into two sub-groups based on the resulting hierarchical tree.
