# Supplementary material for: HyperChIP: identification of hypervariable signals across ChIP-seq or ATAC-seq samples
Source: Genome Biol. 2022 Feb 28;23:62. doi: 10.1186/s13059-022-02627-9 (PMC8883642; doi:10.1186/s13059-022-02627-9)
Supplement: Supplementary file 2 — Additional file 2: Note S1. Statistical simulation. Note S2. Applying HyperChIP without separating proximal and distal regions. S2.1. Evaluating separately the rankings of proximal and distal regions. S2.2. Evaluating the overall rankings of all peak regions. Note S3. Quality control. Note S4. Defining proximal and distal regions. [file 13059_2022_2627_MOESM2_ESM.pdf]

Supplementary Notes for

HyperChIP: identification of hypervariable signals across ChIP-seq or ATAC-seq samples

Haojie Chen[1,2#], Shiqi Tu[1#*], Chongze Yuan[3], Feng Tian[1,2], Yijing Zhang[4], Yihua Sun[3], Zhen Shao[1,2*]

[1]CAS Key Laboratory of Computational Biology, Shanghai Institute of Nutrition and Health, Chinese Academy of Sciences, Shanghai 200031, China.
[2]University of Chinese Academy of Sciences, Beijing 100049, China.
[3]Department of Thoracic Surgery and State Key Laboratory of Genetic Engineering, Fudan University Shanghai Cancer Center, Shanghai 200032, China.
[4]State Key Laboratory of Genetic Engineering, Collaborative Innovation Center of Genetics and Development, Department of Biochemistry, Institute of Plant Biology, School of Life Sciences, Fudan University, Shanghai 200438, China.

#These authors contributed equally to this work.

***Corresponding to:**
Dr. Shiqi Tu
Email: tushiqi@picb.ac.cn

Dr. Zhen Shao
Email: shaozhen@picb.ac.cn

Contents

Note S1. Statistical simulation

Note S2. Applying HyperChIP without separating proximal and distal regions

S2.1 Evaluating separately the rankings of proximal and distal regions

S2.2 Evaluating the overall rankings of all peak regions

Note S3. Quality control

Note S4. Defining proximal and distal regions

References

Note S1. Statistical simulation

The null model of HyperChIP as well as its hypothesis testing procedure has been presented in the main text. Here, we put the associated equations in the following for the convenience of discussion:

$$X_{ij}|\sigma_i^2 \sim N(\mu_i, \gamma\sigma_i^2), \tag{1}$$

$$\frac{1}{\sigma_i^2} \sim \frac{1}{f(\mu_i)} \cdot \frac{\chi_{d_0}^2}{d_0}, \tag{2}$$

$$\frac{\hat{t}_i}{f(\mu_i)} \sim \gamma F_{m-1,d_0}. \tag{3}$$

Note that equation (1) and (2) specify the null model of HyperChIP, while (3) is derived from them.

To test against the alternative hypothesis that region i is an HVR, we make an approximation by replacing μ_i on the left-hand side of (3) with $\hat{\mu}_i$. The resulting statistic (i.e., $\hat{t}_i/f(\hat{\mu}_i)$) is termed the scaled variance, and the right-hand side of (3) is used as its null distribution for deriving the *p*-value. On the one hand, the approximation may introduce additional uncertainty into the test statistic and may, thus, lead to an over-confident *p*-value. On the other hand, we expect the overall *p*-value distribution across regions as well as the specificity of HyperChIP is resistant to the approximation, since the parameter estimation framework of HyperChIP is based on the same approximation as well (see Methods in the main text). More precisely, it is based on the following approximation:

$$\log\frac{\hat{t}_i}{f(\hat{\mu}_i)} \sim \log\gamma + \log F_{m-1,d_0}. \tag{4}$$

In effect, the whole model fitting process treats $\hat{\mu}_i$ as an ordinary covariate for regressing the variances (though it is not strictly non-stochastic) and aims to make the resulting model fit the observed $\hat{t}_i/f(\hat{\mu}_i)$ rather than the unobserved $\hat{t}_i/f(\mu_i)$.

To verify this speculation, we performed a series of statistical simulation in which equation (1) and (2) were used as the data generation process. Formally, the process can be described as follows:

$$\mu_i \sim U(A_{lower}, A_{upper}), \text{ for } i = 1, 2, \dots, n$$

$$\frac{1}{\sigma_i^2} \sim \frac{1}{f(\mu_i)} \cdot \frac{\chi_{d_0}^2}{d_0}, \text{ for } i = 1, 2, \dots, n$$

$$X_{ij} \sim N(\mu_i, \gamma\sigma_i^2), \text{ for } i = 1, 2, \dots, n;\ j = 1, 2, \dots, m$$

where $U(A_{lower}, A_{upper})$ refers to the uniform distribution with A_{lower} and A_{upper} as the lower and upper bounds, respectively. Default parameter settings for this process were as follows:

$$n = 50000,$$

$$A_{lower} = 2,\ A_{upper} = 9,$$

$$f(x) = 0.15 + 6 \cdot 2^{-x},$$

$$d_0 = 28,$$

$$m = 36,$$

$$\gamma = 0.75.$$

These settings were designed to match our empirical observations on the H3K27ac ChIP-seq data set in Table 1.

In each simulation, we used the default settings with modification of at most one parameter, and we inspected the overall *p*-value distribution resulting from the application of HyperChIP:

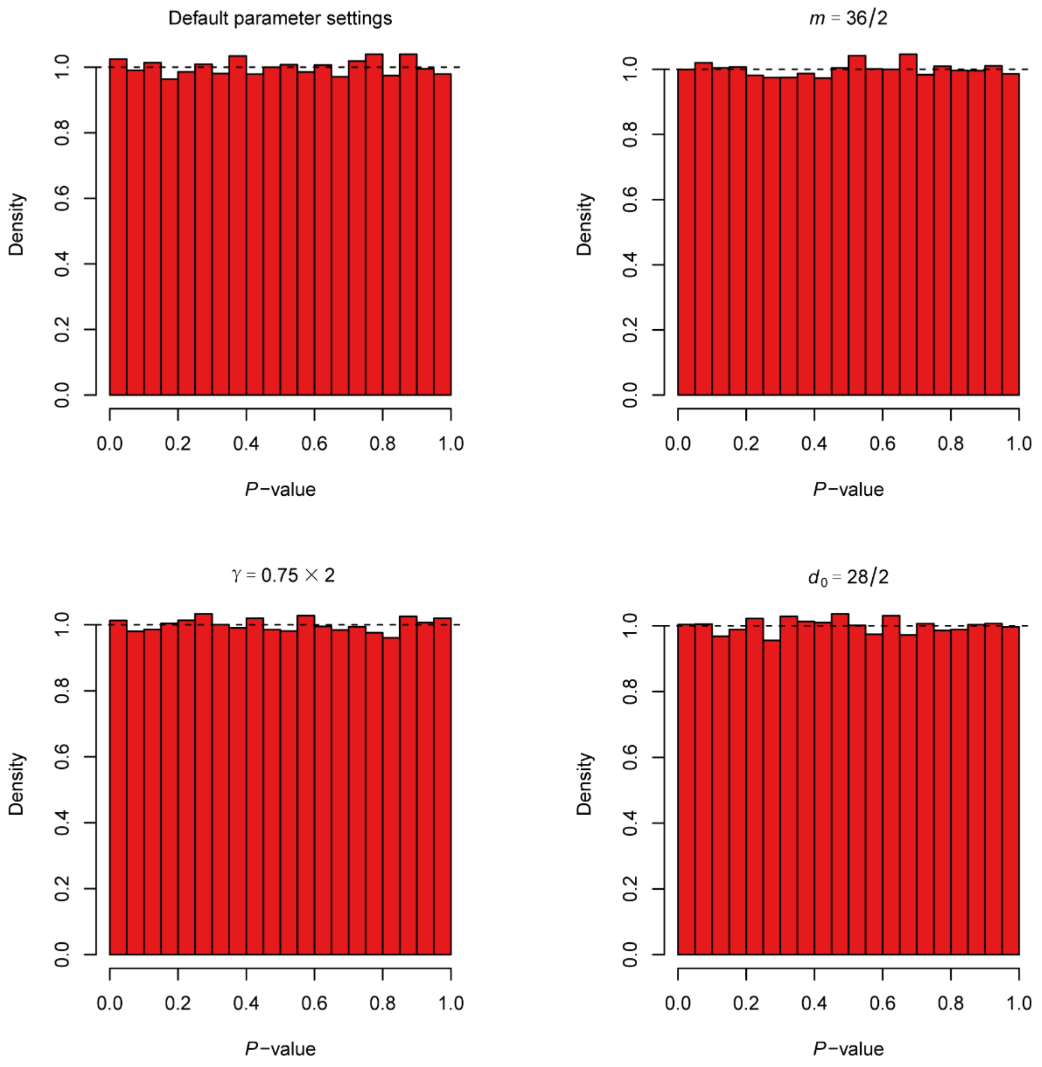

Besides using the default parameter settings, we also tried decreasing the total number of samples, increasing the global signal variability, and decreasing the number of prior degrees of freedom. It can be seen that all the *p*-value distributions are very uniform on [0, 1], indicating HyperChIP is associated with a good control of Type-I error.

Complete R source code for performing the simulations and generating the histograms can be found at https://github.com/tushiqi/MAnorm2/tree/master/utility/code-HyperChIPPaper.

Note S2. Applying HyperChIP without separating proximal and distal regions

For all HyperChIP analyses presented in the main text, we have strictly followed the criterion of separately handling proximal and distal regions. For simplicity, we refer to this analysis strategy as the separated method, and refer to the strategy of processing all peak regions together as the combined method.

S2.1 Evaluating separately the rankings of proximal and distal regions

Both methods have been applied to each of the data sets in Table 1. We first evaluated the rankings of proximal regions by calculating TDPs among top-ranked proximal HVRs, where true discoveries were defined as those linked with HVGs:

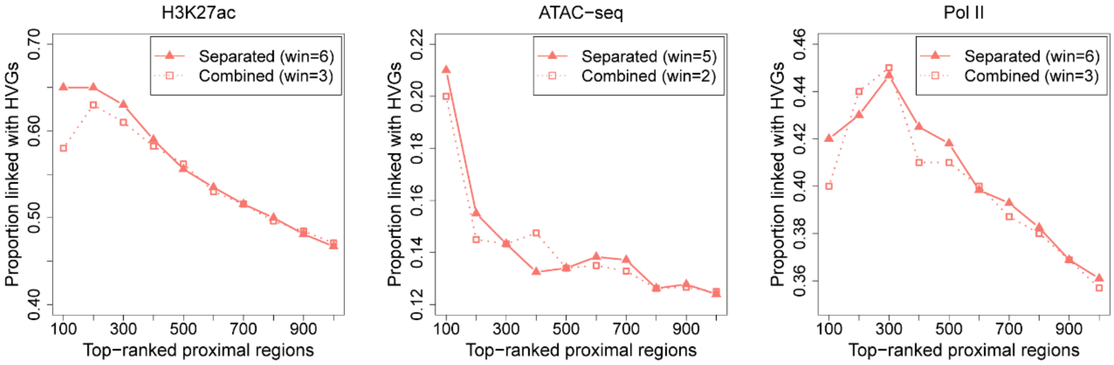

It can be seen that the overall performance of the separated method is better than that of the combined method.

We next repeated the analysis as presented in Figure 2a to evaluate the ability of top-ranked proximal/distal HVRs to distinguish between different classes of samples:

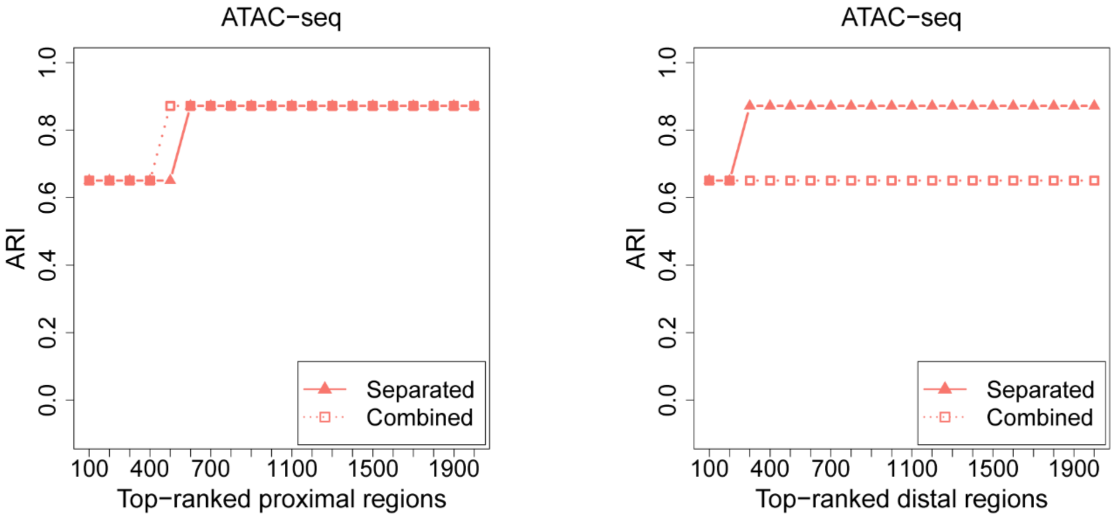

Regarding the rankings of proximal regions, the performance of the combined method was slightly better than that of the separated method, but the latter showed a clear advantage over the former with respect to the rankings of distal regions.

S2.2 Evaluating the overall rankings of all peak regions

We have also performed the above classification analysis by ranking proximal and distal regions together and selecting top-ranked HVRs. For the separated method, we have tried combining the rankings of proximal and distal regions based on the raw *p*-values derived by HyperChIP or the BH-adjusted ones:

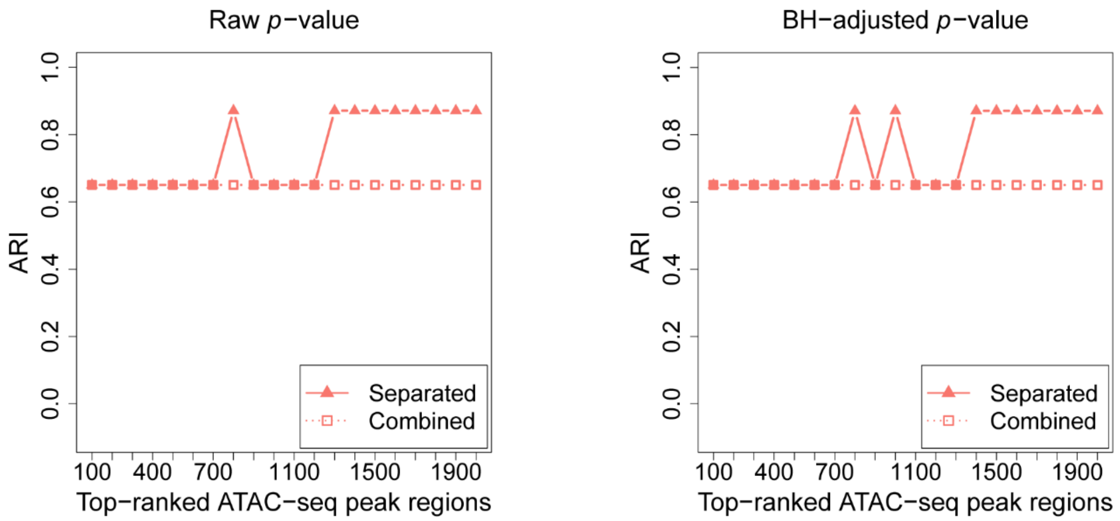

In both cases, the separated method outperformed the combined method.

In the main text, we have showed the t-SNE analysis results from the application of the separated method to the pan-cancer ATAC-seq data set. In the analysis, a BH-adjusted *p*-value cutoff of 0.1 was applied to the selection of both proximal and distal HVRs. In effect, this was equivalent to combining the rankings of proximal and distal regions based on BH-adjusted *p*-values and selecting a certain number of top-ranked HVRs. Here, we applied the combined method to the data set and selected the same total number of top-ranked HVRs for the following t-SNE analysis:

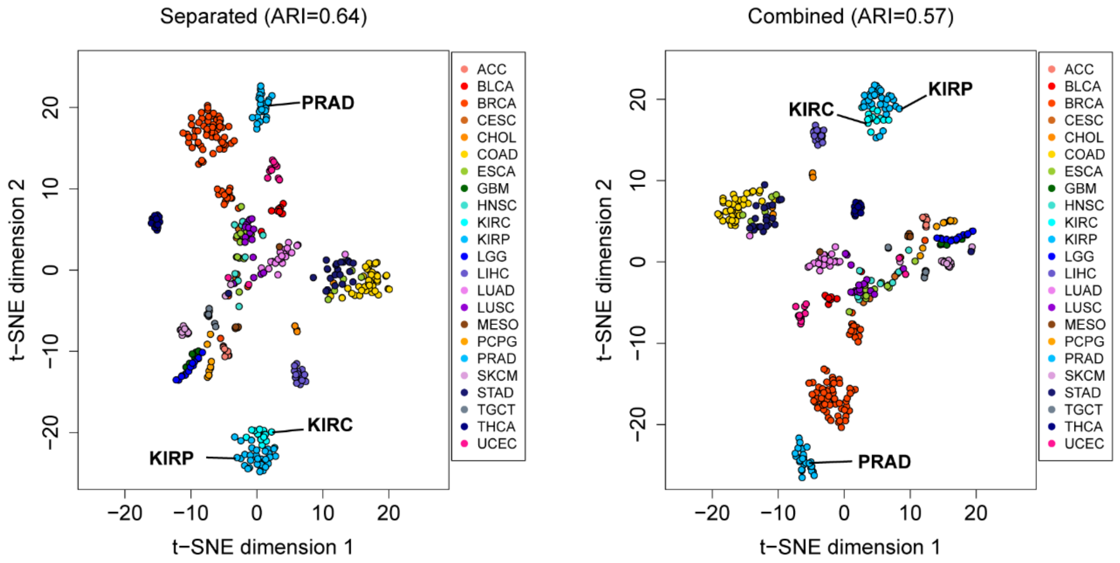

Overall, the two-dimensional t-SNE plots resulting from different methods showed similar patterns. For each method, we also performed a hierarchical clustering of all samples based on the same principal components as used in the t-SNE analysis, and

we classified the samples into 23 sub-groups (the number of cancer types involved) based on the resulting hierarchical tree. It was found that the classification provided by the separated method showed a better agreement with the cancer type labels compared with the combined method (see the titles of the above figures).

Further examination of the t-SNE plots did indicate that the separated method exhibited a better performance in revealing fine structures among the samples. For example, both KIRC and KIRP samples belonged in the kidney carcinoma class. These samples were very close together in both t-SNE plots, but the two cancer types were clearly more distinguishable from one another in the plot generated by the separated method than in the other:

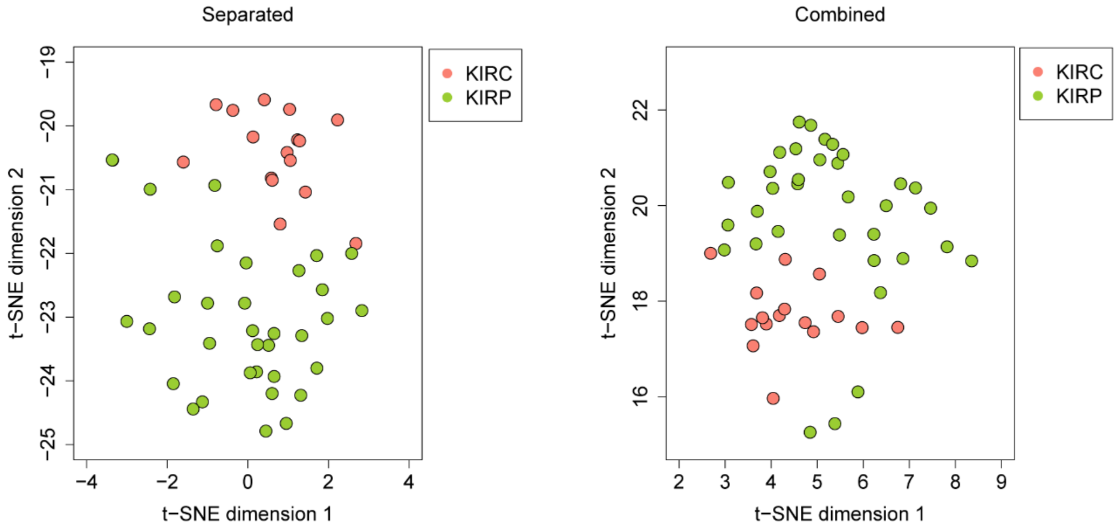

Together, these observations supported the necessity of separately dealing with proximal and distal regions in hypervariable ChIP/ATAC-seq analysis.

Note S3. Quality control

We have assessed the quality of all data sets used in this study but the pan-cancer ATAC-seq one, for which only a count table was available. The statistics of primary interest were peak number and FRiP:

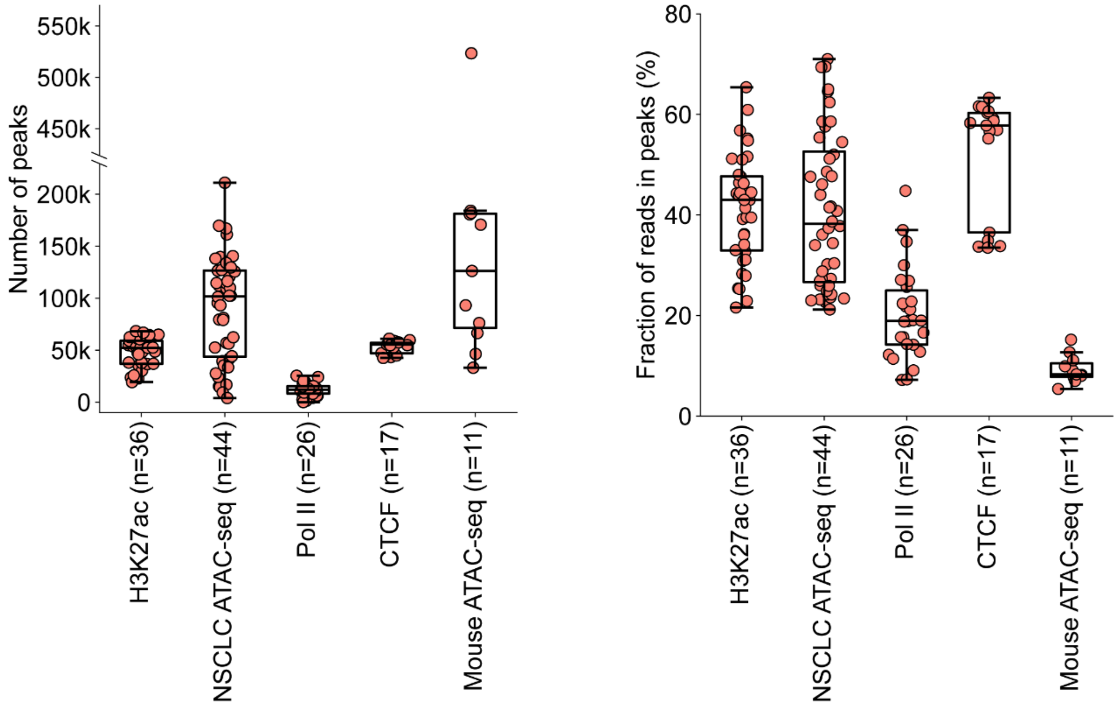

It can be seen that the distribution of peak numbers varies significantly across the data sets. In particular, the peak number associated with an ATAC-seq sample is typically larger than that with a ChIP-seq sample, and the latter largely depends on the specific TF or histone modification targeted in the experiment. In comparison, the distribution of FRiPs is more comparable across the data sets. Compared with the other four data sets, the mouse ATAC-seq data set is associated with much lower FRiPs because of the low input materials obtained from preimplantation embryos for the ATAC-seq experiments [1]. The CTCF ChIP-seq samples form two sub-groups that are associated with distinct FRiPs, and we have confirmed that there was no clear association between the group labels of samples and their populations of origin (the lower-FRiP group consisted of one Caucasian individual and four Yoruban individuals).

Since none of the data sets was associated with outlier samples that had extremely low peak number or FRiP, we basically retained every sample but only filtered out the NSCLC ATAC-seq samples with less than 40k peaks, which was based on our previous experience in analyzing ATAC-seq data sets from cancer studies. As a result, 10 NSCLC ATAC-seq samples were removed, and all the associated analyses presented in the main text were based on the remaining 34 samples. For the sake of rigor, we repeated the related benchmarking analyses without filtering out any NSCLC ATAC-seq samples:

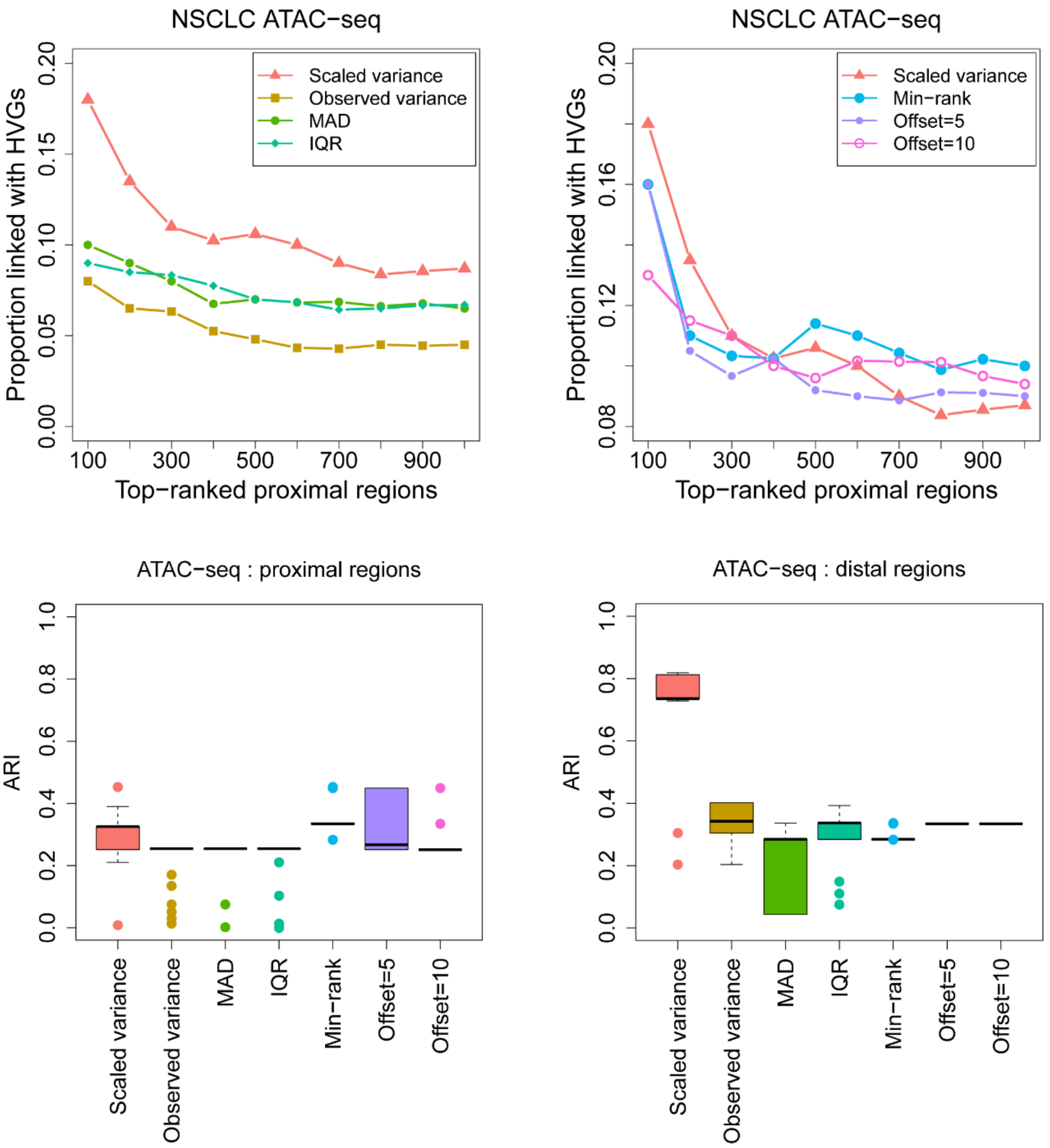

With respect to the consistency with HVGs, HyperChIP clearly outperformed the methods that do not consider the mean-variability dependence, and was comparable to the other methods that consider it. Similar relative performance was also observed when we evaluated the classifications based on top-ranked proximal HVRs identified by different methods. For the classifications based on top-ranked distal HVRs, however, the performance of HyperChIP was dramatically better than all the other methods.

Note S4. Defining proximal and distal regions

The primary reason for which we propose the separation of proximal and distal regions in hypervariable ChIP/ATAC-seq analysis is that the global ChIP/ATAC-seq signal variability in distal regions is typically higher than that in proximal regions (Fig. 8), since the activity of distal regulatory elements is much more variable across cellular contexts and human individuals than is gene expression [2-4], while the activity of proximal ones is tightly connected with the expression of nearby genes.

To determine a distance cutoff in an objective manner for defining proximal and distal regions, we examined, for each data set in Table 1, the Pearson correlation coefficient (PCC) between the ChIP/ATAC-seq signal intensities in each peak region and the expression of the nearest gene ($\log_2$-CPM values calculated from RNA-seq samples):

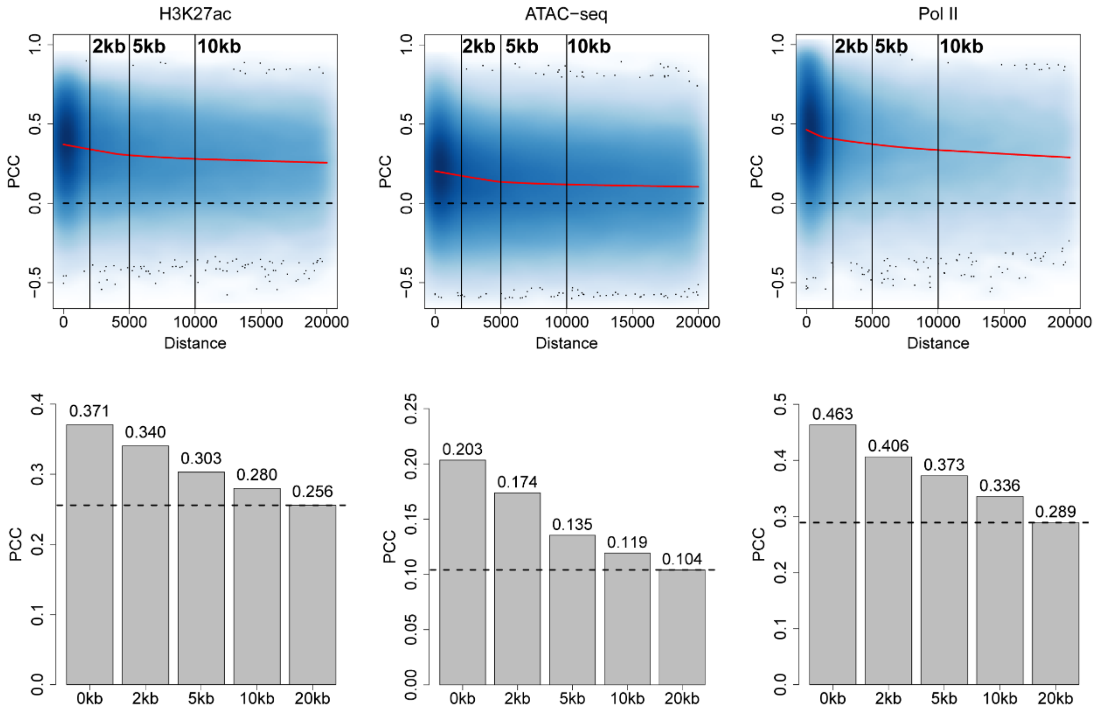

Here, the distance associated with each peak region is from its near end to the nearest transcription start site (TSS), and the distance is considered as 0 if the TSS is within the peak region. The red curves are fitted by applying LOWESS (locally-weighted polynomial regression), and the bar plots are created based on the fitted PCCs. It can be seen that, for each data set, the peak regions occupying a TSS achieve the highest fitted PCC, which gradually decreases as the peak regions become further away from TSSs.

For all the data sets, we noticed that the peak-gene association was still strong up to 5kb away from TSSs. In particular, the fitted PCC at 5kb was within 0.1 of the maximum value for each data set. Moreover, for both the H3K27ac ChIP-seq and ATAC-seq data sets, the fitted PCC curves had break points near 5kb (the curve associated with the Pol II ChIP-seq data set had a break point less than 2kb). We

therefore chose 5kb as the distance cutoff for separating proximal and distal regions.

For the sake of rigor, we also tried using 2kb as the distance cutoff and repeated the benchmarking analyses as presented in Figure 1c, d and Figure 2. We first examined the consistency between top-ranked proximal HVRs and HVGs:

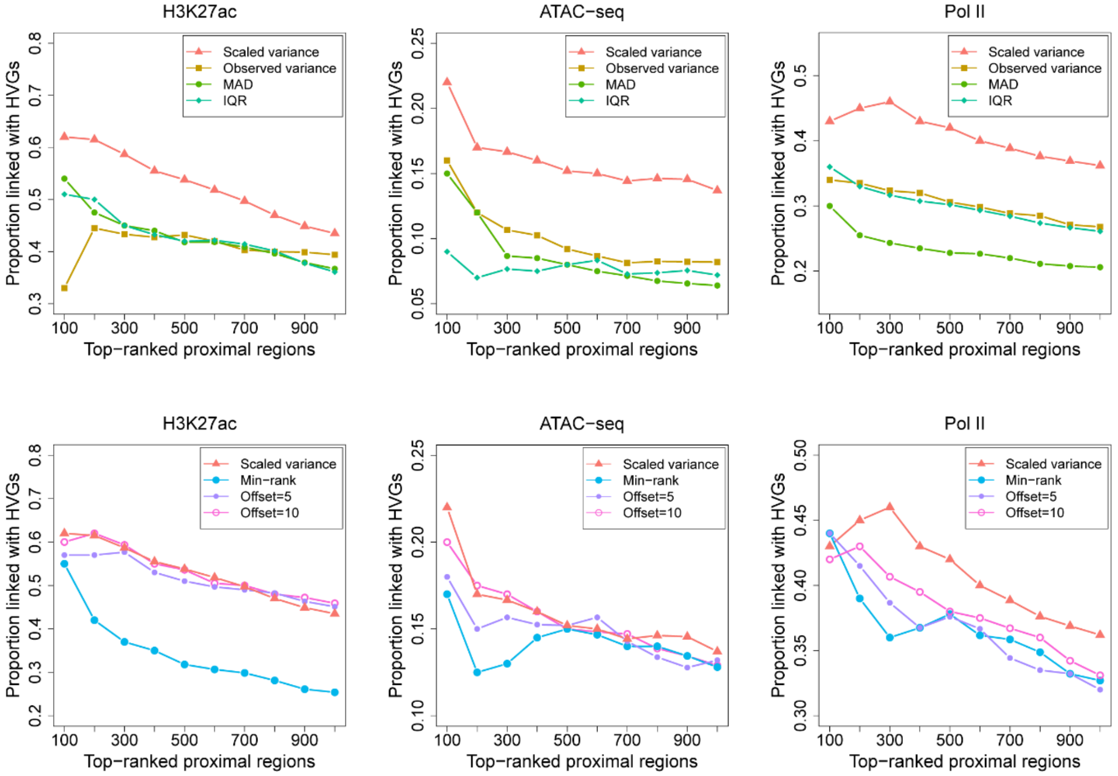

The results were similar as before: HyperChIP clearly outperformed the methods that do not consider the mean-variability dependence, and it performed better or as well compared with the other methods. We then evaluated the classifications of the NSCLC ATAC-seq samples based on top-ranked proximal/distal HVRs:

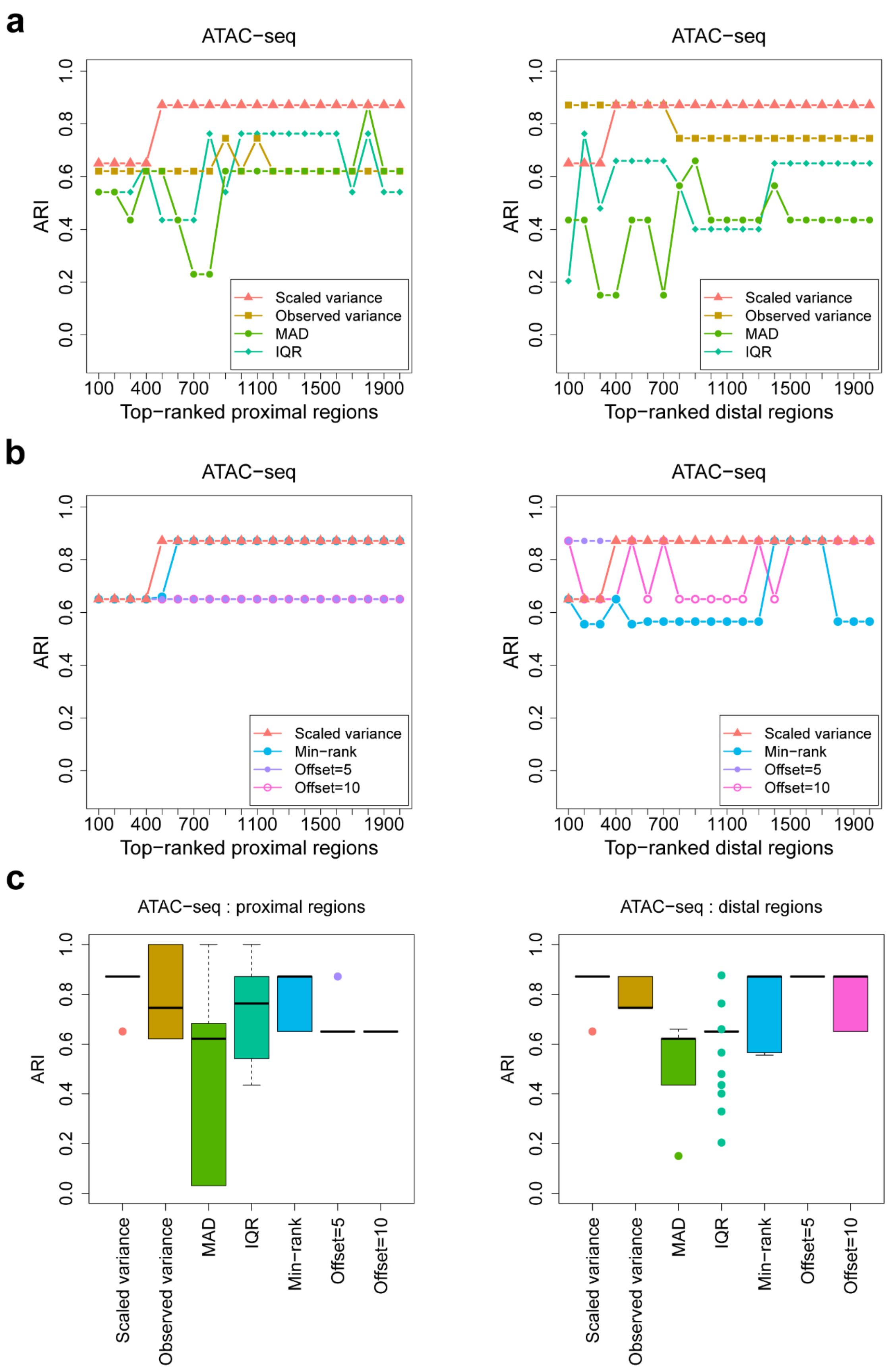

With respect to the rankings of proximal HVRs, HyperChIP clearly outperformed the methods that consider the mean-variability dependence. For the methods that do not

consider it, their performance was occasionally better than HyperChIP but was far from stable. With respect to the rankings of distal HVRs, the performance of HyperChIP was comparable to that of the offset=5 method, and both of them clearly outperformed all the other methods.

References

1. Wu J, Huang B, Chen H, Yin Q, Liu Y, Xiang Y, Zhang B, Liu B, Wang Q, Xia W, et al: **The landscape of accessible chromatin in mammalian preimplantation embryos.** *Nature* 2016, **534:**652-657.
2. Ernst J, Kheradpour P, Mikkelsen TS, Shoresh N, Ward LD, Epstein CB, Zhang X, Wang L, Issner R, Coyne M, et al: **Mapping and analysis of chromatin state dynamics in nine human cell types.** *Nature* 2011, **473:**43-49.
3. Kasowski M, Kyriazopoulou-Panagiotopoulou S, Grubert F, Zaugg JB, Kundaje A, Liu Y, Boyle AP, Zhang QC, Zakharia F, Spacek DV, et al: **Extensive variation in chromatin states across humans.** *Science* 2013, **342:**750-752.
4. Heinz S, Romanoski CE, Benner C, Glass CK: **The selection and function of cell type-specific enhancers.** *Nat Rev Mol Cell Biol* 2015, **16:**144-154.
