# Supplementary material for: HyperChIP: identification of hypervariable signals across ChIP-seq or ATAC-seq samples
Source: Genome Biol. 2022 Feb 28;23:62. doi: 10.1186/s13059-022-02627-9 (PMC8883642; doi:10.1186/s13059-022-02627-9)
Supplement: Supplementary file 3 — Additional file 3: Table S1. Top 20 GO terms enriched from the genes linked with the significant proximal LVRs identified for the H3K27ac ChIP-seq data set. Table S2. Top 20 GO terms enriched from the genes linked with the significant proximal HVRs identified for the H3K27ac ChIP-seq data set. Table S3. Cancer types involved in the TCGA ATAC-seq data set. Note that in TCGA studies the abbreviation CESC refers to both cervical squamous cell carcinoma and endocervical adenocarcinoma, but all the 4 CESC patients in this data set belong in the former. Note also that the 18 ESCA patients in this data set consist of 12 ESSC (esophageal squamous cell carcinoma) and 6 ESAD (esophageal adenocarcinoma) cases, which belong in the SC and DIAD classes, respectively. SC, squamous cell carcinoma; DIAD, digestive adenocarcinoma. [file 13059_2022_2627_MOESM3_ESM.pdf]

GO term	$-\mathrm{Log}_{10}$(*p*-value)
histone lysine demethylation (GO:0070076)	4.52
tRNA modification (GO:0006400)	4.13
RNA catabolic process (GO:0006401)	4.01
histone H3 acetylation (GO:0043966)	3.90
histone acetylation (GO:0016573)	3.84
cellular macromolecule catabolic process (GO:0044265)	3.70
histone H3-K4 demethylation (GO:0034720)	3.32
energy coupled proton transport, down electrochemical gradient (GO:0015985)	3.28
cellular response to gamma radiation (GO:0071480)	3.00
purine ribonucleoside monophosphate biosynthetic process (GO:0009168)	2.97
DNA metabolic process (GO:0006259)	2.93
nucleobase-containing compound catabolic process (GO:0034655)	2.80
purine-containing compound biosynthetic process (GO:0072522)	2.78
RNA metabolic process (GO:0016070)	2.72
regulation of generation of precursor metabolites and energy (GO:0043467)	2.72
genetic imprinting (GO:0071514)	2.64
NADH metabolic process (GO:0006734)	2.64
cellular response to ionizing radiation (GO:0071479)	2.61
cristae formation (GO:0042407)	2.53
ribonucleoside bisphosphate biosynthetic process (GO:0034030)	2.49

Table S1. Top 20 GO terms enriched from the genes linked with the significant proximal LVRs identified for the H3K27ac ChIP-seq data set.

GO term	$-Log_{10}$(*p*-value)
positive regulation of transcription from RNA polymerase II promoter (GO:0045944)	6.75
regulation of transcription from RNA polymerase II promoter (GO:0006357)	6.62
cartilage development (GO:0051216)	6.12
epidermis development (GO:0008544)	6.12
surfactant homeostasis (GO:0043129)	5.65
chemical homeostasis within a tissue (GO:0048875)	5.65
positive regulation of transcription, DNA-templated (GO:0045893)	5.52
kidney development (GO:0001822)	4.90
negative regulation of multicellular organismal process (GO:0051241)	4.71
chondrocyte differentiation (GO:0002062)	4.44
regulation of cartilage development (GO:0061035)	4.38
renal system development (GO:0072001)	4.35
lung development (GO:0030324)	4.18
collecting duct development (GO:0072044)	3.99
renal absorption (GO:0070293)	3.86
endochondral bone morphogenesis (GO:0060350)	3.86
endochondral ossification (GO:0001958)	3.64
positive regulation of epithelial cell differentiation (GO:0030858)	3.52
negative regulation of transcription, DNA-templated (GO:0045892)	3.48
negative regulation of cell differentiation (GO:0045596)	3.43

Table S2. Top 20 GO terms enriched from the genes linked with the significant proximal HVRs identified for the H3K27ac ChIP-seq data set.

Cancer type	Abbreviation	Annotation	Cohort size
Adrenocortical carcinoma	ACC		9
Bladder Urothelial Carcinoma	BLCA	SC	10
Breast invasive carcinoma	BRCA		75
Cervical squamous cell carcinoma and endocervical adenocarcinoma	CESC	SC	4
Cholangiocarcinoma	CHOL		5
Colon adenocarcinoma	COAD	DIAD	41
Esophageal carcinoma	ESCA	SC or DIAD	18
Glioblastoma multiforme	GBM	Brain cancer	9
Head and Neck squamous cell carcinoma	HNSC	SC	9
Kidney renal clear cell carcinoma	KIRC	Kidney carcinoma	16
Kidney renal papillary cell carcinoma	KIRP	Kidney carcinoma	34
Brain Lower Grade Glioma	LGG	Brain cancer	13
Liver hepatocellular carcinoma	LIHC		17
Lung adenocarcinoma	LUAD		22
Lung squamous cell carcinoma	LUSC	SC	16
Mesothelioma	MESO		7
Pheochromocytoma and Paraganglioma	PCPG		9
Prostate adenocarcinoma	PRAD		26
Skin Cutaneous Melanoma	SKCM		13
Stomach adenocarcinoma	STAD	DIAD	21
Testicular Germ Cell Tumors	TGCT		9
Thyroid carcinoma	THCA		14
Uterine Corpus Endometrial Carcinoma	UCEC		13

Table S3. Cancer types involved in the TCGA ATAC-seq data set. Note that in TCGA studies the abbreviation CESC refers to both cervical squamous cell carcinoma and endocervical adenocarcinoma, but all the 4 CESC patients in this data set belong in the former. Note also that the 18 ESCA patients in this data set consist of 12 ESSC (esophageal squamous cell carcinoma) and 6 ESAD (esophageal adenocarcinoma) cases, which belong in the SC and DIAD classes, respectively. SC, squamous cell carcinoma; DIAD, digestive adenocarcinoma.
